# The Actomyosin Machinery Is Required for *Drosophila* Retinal Lumen Formation

**DOI:** 10.1371/journal.pgen.1004608

**Published:** 2014-09-18

**Authors:** Jing Nie, Simpla Mahato, Andrew C. Zelhof

**Affiliations:** Department of Biology, Indiana University, Bloomington, Indiana, United States of America; Stanford University, United States of America

## Abstract

Multicellular tubes consist of polarized cells wrapped around a central lumen and are essential structures underlying many developmental and physiological functions. In *Drosophila* compound eyes, each ommatidium forms a luminal matrix, the inter-rhabdomeral space, to shape and separate the key phototransduction organelles, the rhabdomeres, for proper visual perception. In an enhancer screen to define mechanisms of retina lumen formation, we identified Actin5C as a key molecule. Our results demonstrate that the disruption of lumen formation upon the reduction of *Actin5C* is not linked to any discernible defect in microvillus formation, the rhabdomere terminal web (RTW), or the overall morphogenesis and basal extension of the rhabdomere. Second, the failure of proper lumen formation is not the result of previously identified processes of retinal lumen formation: Prominin localization, expansion of the apical membrane, or secretion of the luminal matrix. Rather, the phenotype observed with *Actin5C* is phenocopied upon the decrease of the individual components of non-muscle myosin II (MyoII) and its upstream activators. In photoreceptor cells MyoII localizes to the base of the rhabdomeres, overlapping with the actin filaments of the RTW. Consistent with the well-established roll of actomyosin-mediated cellular contraction, reduction of MyoII results in reduced distance between apical membranes as measured by a decrease in lumen diameter. Together, our results indicate the actomyosin machinery coordinates with the localization of apical membrane components and the secretion of an extracellular matrix to overcome apical membrane adhesion to initiate and expand the retinal lumen.

## Author Summary

Biological tubes are integral units of tissues and organs such as lung, kidney, and the cardiovascular system. The fundamental design of tubes involves a central lumen wrapped by a sheet of cells. To function properly, the tubes require a precise genetic control over their creation, the diametric growth and maintenance of the lumen during development. In the fruit fly, *Drosophila melanogaster*, the photoreceptor cells of the eye form a tubular structure. The formation of the retinal lumen is critical for separating and positioning the light sensing organelles of each photoreceptor cell to achieve visual sensitivity. In an effort to investigate the mechanisms of *Drosophila* retinal lumen formation, we identified a contractile machinery that was present at the apical portion of photoreceptor cells. Our data is consistent with the idea that a contractile force contributes to the initial separation of the juxtaposed apical membranes and subsequent enlargement of the luminal space. Our work suggests that building a biological tube requires not only an extrinsic pushing force provided by the growing central lumen, but also a cell intrinsic pulling force powered by contraction of cells lining the lumen. Our findings expand and demonstrate the coordination of several molecular mechanisms to generate a tube.

## Introduction

Multicellular tubes are fundamental structures required for the transport of gases, liquids, or cells and are necessary for the generation and function of tissues and organs such as lung, kidney, blood vessels, neural tubes, and mammary gland. The main feature of a tubular network is a luminal space lined by apical membranes of polarized epithelial or endothelial cells. To construct a functional tube, there needs to be mechanisms to first generate a luminal space and then regulate the expansion and determination of the final diametrical size of the lumen. Cells utilize multiple pathways to organize themselves to form an initial tubular network (reviewed in [1]–[3]) and likewise diametric luminal growth appears to be under precise genetic control [4]. To date lumen growth has been characterized as a process of directed and regulated apical secretion of components into a central space and a reorganization of the apical membrane. Secretion likely provides a mechanical expansion force that drives the diametrical growth of the tube lumen [5]–[7]. Secreted components can include solid extracellular matrices of proteoglycans and collagens. Increase in lumen size can also be achieved by increasing the osmotic lumen pressure via ion pumps and channels [8]–[10]. Furthermore, the fusion of secretory vesicles with apical plasma membranes often changes the cells apical domain antigens, which in return drive the expansion of apical membrane permitting an increase in the diameter of the lumen [4], [11], [12].

The *Drosophila* compound eye provides an ideal model system to study lumen formation. The *Drosophila* eye consists of approximately 800 individual units known as ommatidia. In each ommatidium, a tubular structure is generated by the concerted efforts of the eight photoreceptors. Over a period ∼60 hours (h), the reorganization of the photoreceptor apical membranes drives a dramatic morphogenesis from a single epithelial sheet to a single tube containing eight cells surrounding a central lumen matrix, termed the inter-rhabdomeral space (IRS) [13]. Furthermore, each photoreceptor projects its corresponding light sensing organelle, the rhabdomere, within the luminal space. Consequentially, the IRS is required to shape the rhabdomeres and optically position each rhabdomere to achieve proper visual sensitivity [14], [15].

Genetic dissection of retinal lumen formation has provided key insights into fundamental questions about lumen formation, such as the mechanism through which adherent juxtaposed membranes separate. To date *Drosophila* retinal lumen formation is known to be dependent on secretion of an extracellular matrix [16], [17] and a concurrent steric hindrance of Chaoptin (Chp) based adhesion [17]. The major constituent of the IRS matrix is the proteoglycan protein Eyes shut (EYS), which is also known as Spacemaker. Loss of EYS results in a complete failure of the IRS to form [16], [17]. Nonetheless, secretion of EYS is not sufficient for the generation of a continuous lumen. In addition to being secreted, EYS must be localized around the developing apical rhabdomeres through an interaction with the five-pass transmembrane protein Prominin (Prom) [17], [18]. In the absence of Prom, the lumen space is present but not continuous and the residual fusion between rhabdomeres is the result of the adhesion between the rhabdomeric apical membranes mediated by the GPI-anchored membrane protein Chp [17], [19], [20].

Although these previous results demonstrate the interplay between secretion and adhesion, here we reveal an additional mechanism contributing to retinal lumen formation and expansion. Our results implicate that actin and non-muscle myosin II (MyoII) generate a contractile force at the apical domain of photoreceptors to separate the initial juxtaposed membranes. Rhabdomere adhesion is enhanced upon reduction of components of the actomyosin complex or its upstream activators in our sensitized genetic background. Additionally, knockdown of MyoII in a wild-type background led to a narrower lumen space. Temporal profiling of the key molecules for retinal lumen formation indicates the actomyosin complex is the first to localize to the apical surface followed by Prom and EYS. Thus the actomyosin machinery would be providing an initial apical based tension on the Chp based juxtaposed membranes, assisting the initiation and expansion of subsequently deposited extracellular matrix represented by EYS. All together, our genetic analysis has revealed an unappreciated facet of lumen formation and outlined the temporal steps and coordination required to achieve membrane separation.

## Results

### Reduction of *Actin5C* enhances rhabdomere adhesion in an *eys*, *prom* trans-heterozygote mutant background

**Figure 1:**
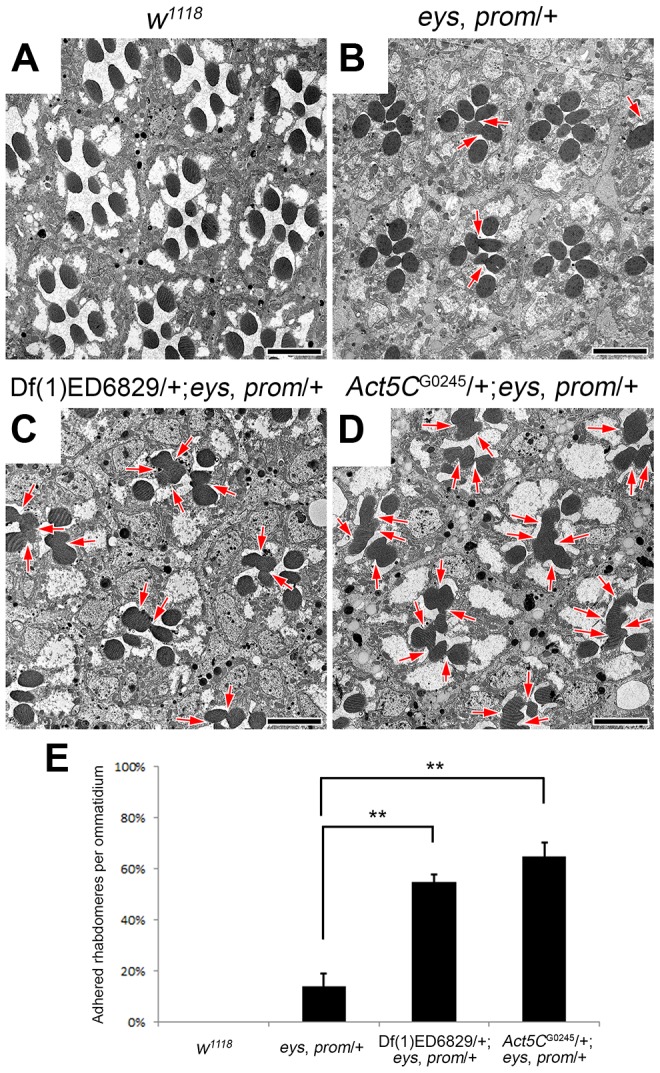
Reduction of *Act5C* dosage enhances rhabdomere adhesion. (A–D) Transmission electron micrographs of adult *Drosophila* ommatidia. (A) *w^1118^*, wild type. (B) *eys*, *prom*/+. (C) Df(1)ED6829/+; *eys*, *prom*/+. The deficiency removes genomic region 5C7-5F3. (D) *Act5C* ^G0245^/+; *eys*, *prom*/+. (E) Quantitative analysis of rhabdomere fusion seen in (A–D). Values represent mean ± SEM. **P<0.01 compared with *eys*, *prom*/+. Arrows indicate the incomplete separation between rhabdomeres. Scale bar, 5 µm.

The separation of rhabdomeres and formation of the retinal lumen, the IRS, within each ommatidium depends on the fine balance between an adhesion force, provided by Chp [19]–[22], and an expansion force provided by EYS [16], [17] and Prom [17]. When one copy of both *eys* and *prom* are removed there is a partial failure to form a continuous open IRS, exhibited by the presence of juxtaposed rhabdomeres (compare Figure 1 A,B). However, the phenotype is not fully penetrant (Figure 1B,E) and manipulating the levels Prom, EYS, or Chp can modulate the appearance of a continuous retinal lumen [17]. Utilizing this sensitized genetic background, we performed a genetic screen to identify other genes required for the formation of the IRS. Defined genomic deletions were introduced into the *eys*, *prom* trans-heterozygote (EP-TH) background and scored for the ability to enhance the EP-TH adhesion phenotype and thus *Drosophila* eyes were screened for the loss of the deep pseudopupil [23]. From our screen, we identified deletion Df(1)ED6829 (www.flybase.org) as a potential candidate. Transmission electron microscopy (TEM) analysis confirmed that rhabdomere fusion was significantly enhanced upon the inclusion of the deletion in the double heterozygous background. The addition of Df(1)ED6829 in the EP-TH resulted in an 41% increase in rhabdomere fusion (Figure 1 C,E).

Analysis of overlapping deletions and testing of individual mutants for genes within the deleted interval mapped the responsible locus to *Actin5C* (*Act5C*), as alleles of *Act5C* as well as mild RNAi knockdown of *Act5C* in the EP-TH background completely phenocopied the phenotype observed with the deficiency (Figure 1D, E and Figure S1). In *Drosophila* there are six actin genes: *Act5C* and *Act42A* are ubiquitously expressed, while *Act57B*, *Act79B*, *Act87E*, and *Act88F* are muscle-specific [24]–[26]. To test the specificity of our enhancement, we re-examined deletions that individually remove each actin gene. TEM analyses of the corresponding deficiencies for the other five actin genes did not show any enhancement of fused rhabdomeres in the EP-TH background (Figure S2). These data suggested that *Act5C* was a specific and key component in generating the retinal lumen.

### The genetic reduction of *Act5C* does not alter the dynamics of rhabdomere morphogenesis or structure

Photoreceptors are dependent upon actin for overall structure and integrity. In particular, the microvilli of the rhabdomeres contain an actin cytoskeleton core [27] and the rhabdomeres are supported by the actin-based structure the rhabdomere terminal web (RTW) [28]. The RTW is also responsible for directing delivery of molecules to the rhabdomere [28], [29]. Thus, the enhancement of rhabdomere fusion observed in the triple heterozygote may be an indirect result of disruption of photoreceptor actin based structures. To test this possibility, we examined the ability of the rhabdomere to extend the entire length of the photoreceptor cell body to the cone cell plate [30]. We found the removal of one genetic copy of *Act5C* in a wild type or EP-TH background does not affect the ability of the rhabdomeres to extend the entire length (Figure S3). Nor did we observe any change in rhabdomere diameter, an indication that microvilli formation and extension is normal (Figure 1). With respect to the formation of the RTW, heterozygous mutation of *Act5C* does not affect the localization of Moesin (Moe) (Figure S4) and RNAi knockdown of *moe* in the EP-TH background led to rhabdomere degeneration as observed in a *moe* mutant [28]. Thus our results do not indicate that the increase in juxtaposed rhabdomeres was the result of a general defect in the actin-based structures of photoreceptors.

### The reduction of Actin5C does not affect spatial localization of EYS, Prominin, or Crumbs

**Figure 2:**
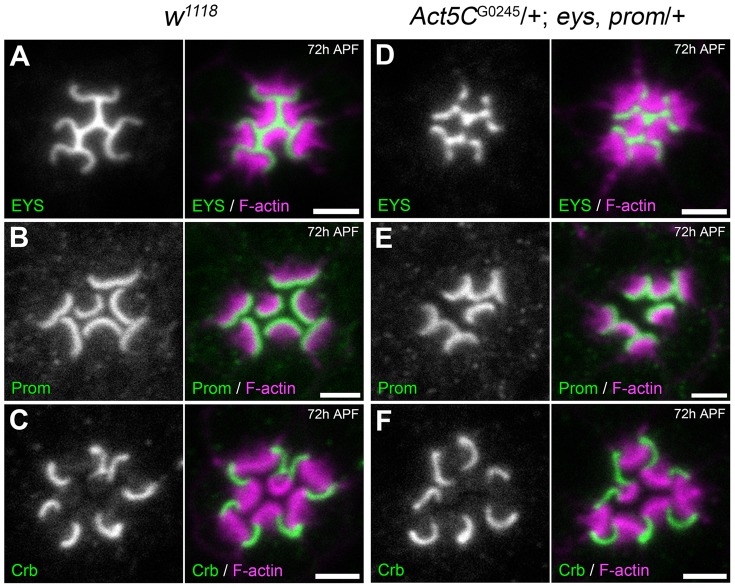
Localization of Prominin, EYS, and Crumbs in the *Act5C*/+; EP-TH genetic background. Confocal immunofluorescence micrographs of wild type (A–C) and *Act5C* ^G0245^/+; *eys*, *prom*/+ (D–F) ommatidium at 72 h APF stained with: (A,D) EYS and F-actin. (B,E) Prominin (Prom) and F-actin. (C,F) Crumbs (Crb) and F-actin. Scale bar, 2 µm.

What is the specific primary defect upon the genetic reduction *Act5C*? Besides structural support, the actin cytoskeleton provides tracks in the cell to allow intracellular transportation of membrane and non-membrane-bound cargos [31], [32]. Furthermore, in mammalian photoreceptors the mammalian Prominin ortholog, Prominin1, directly interacts with actin; actin co-immunoprecipitates with Prominin1 and the binding is attenuated by the Prominin1 R373C mutation [33]. Thus it is conceivable that the increase in rhabdomere fusion upon the genetic reduction of Actin results from either mislocalized or missing apical components or defects in secretion of EYS. We addressed these possibilities by first surveying whether there was any difference in the spatial localization pattern of Prom and EYS in the *Act5C/+*; EP-TH background. We did not observe any detectable decrease or mislocalized EYS (Figure 2 A,D) or Prom (Figure 2 B,E). In addition to EYS and Prom, the transmembrane protein Crumbs (Crb) has also been implicated in rhabdomere separation and *Drosophila* salivary gland lumen formation [11], [21], [34], [35]. In photoreceptors, Crb localizes to the stalk membrane, the portion of the apical membrane void of microvilli, and is critical for regulating the length of the stalk membrane. In *crb* mutant flies neighboring adjacent rhabdomeres remain juxtaposed [21], [34]–[37] implying the shortening of the stalk membrane permitted adjacent rhabdomeres to remain together. In the *Act5C/+*; EP-TH background we did not observe any visible alteration in Crb localization (Figure 2 C,F). Taken together these results implied that the increase in juxtaposed rhabdomeres, upon the decrease in *Act5C* genetic dosage, was not due to an obvious mislocalization of known retinal lumen formation proteins.

### Loss of non-muscle myosin II results in a discontinuous retinal lumen

To identify the mode of action responsible for the loss of a continuous luminal space in *Act5C/+*; EP-TH, we next examined the role of the actomyosin network. Actin is well known to interact with non-muscle myosin II (MyoII) to generate a contraction force to induce cellular morphological changes (for reviews see [38]–[40]). Previous studies in *Drosophila* photoreceptors have demonstrated a role of the actomyosin complex in regulating photoreceptor cell body position, adherens junction formation and apical contraction within the morphogenetic furrow [41]–[43]. Therefore, a plausible explanation was that the reduction of *Act5C* dosage decreased a contractile force in photoreceptors resulting in inability of the apical membranes to pull away from each other during the initial phase of lumen formation.

**Figure 3:**
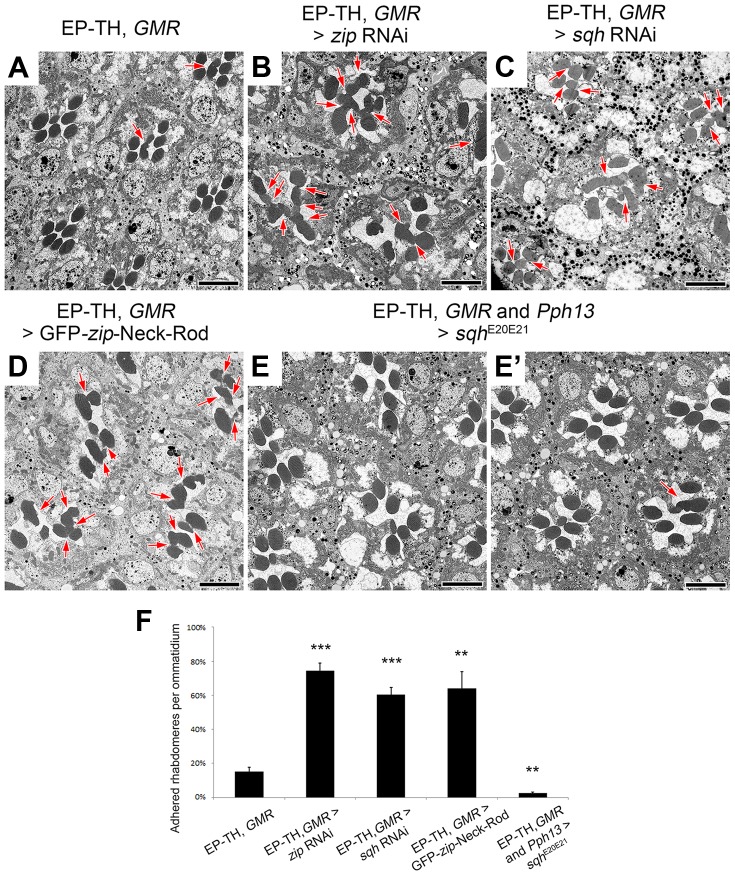
Non-muscle myosin II is involved in retinal lumen formation. (A–E′) Transmission electron micrographs of adult *Drosophila* ommatidia. (A) *eys*, *prom*, *GMR*-GAL4/+. (B) *eys*, *prom*, *GMR*-GAL4/+; UAS-*zip* RNAi/+. (C) *eys*, *prom*, *GMR*-GAL4/+; UAS-*sqh* RNAi/+. (D) *eys*, *prom*, *GMR*-GAL4/+; UAS-GFP-*zip*-Neck-Rod/+. (E and E′) *eys*, *prom*, *GMR*-GAL4/+; *Pph13*-Gal4/UAS-*sqh* ^E20E21^. (F) Quantitative analysis of rhabdomere fusion seen in (A–E′). Values represent mean ± SEM. *** *P*<0.001, **P<0.01 compared with *eys*, *prom*, *GMR*-Gal4/+. Arrows indicate adhesion between rhabdomeres. Scale bar, 5 µm.

Non-muscle myosin II molecules are hexamers comprised of three pairs of subunits [44]: two heavy chains, encoded by *zipper* (*zip*), two regulatory light chains, *spaghetti squash* (*sqh*), and two essential light chains [45]–[47]. Unlike the loss of one copy of *Act5C*, a deletion that removes *zip* as well as specific *zip* alleles did not enhance the EP-TH phenotype. Nonetheless, only removing one genetic dosage of *zip* may not have been sufficient to lower protein levels. In an attempt to further reduce Zip protein levels we employed RNAi against *zip*. The ability of *zip* RNAi to decrease Zip protein levels was confirmed by immunofluorescence detection in 48 h after puparium formation (APF) pupal eyes with utilization of *zip* RNAi flip-out clones (Figure S5 A,B). Upon RNAi knockdown, we observed a significant increase in fused rhabdomeres in the EP-TH background that mimicked the loss of one copy of *Act5C* (Figure 3 A,B,F). To demonstrate phenotypic specificity, co-expression of UAS-GFP-*zip* along with UAS-*zip-*RNAi rescued the rhabdomere adhesion phenotype back to the EP-TH background levels (Figure S5 E,F). To further eliminate the possibility of RNAi off-target effects, we assayed the ability of a dominant-negative (DN) form of Zip (UAS-GFP-*zip*-Neck-Rod) [48], [49] to enhance rhabdomere fusion. We obtained a significant enhancement with the expression of the dominant-negative form of Zip in the EP-TH background (Figure 3 D,F). Similar to the MyoII heavy chain Zip, reduction of the myosin regulatory light chain, Sqh, also displayed an essential role in retinal lumen formation. RNAi knockdown of *sqh*, confirmed by immunofluorescence in flip-out clones (Figure S5 C,D), substantially enhanced the presence of juxtaposed rhabdomeres in the EP-TH background (Figure 3 C,F). In contrast, the co-expression of a constitutively active form of Sqh (UAS-*sqh* ^E20E21^) [50] with the *sqh* RNAi construct reduced the rhabdomere enhancement back to EP-TH levels (Figure S5 G,H). More importantly, the expression of the constitutively active Sqh alone in the EP-TH background rescued the rhabdomere adhesion. The frequency of observed juxtaposed rhabdomeres was significantly reduced from 15% per ommatidium to 3% (Figure 3 E,F).

**Figure 4:**
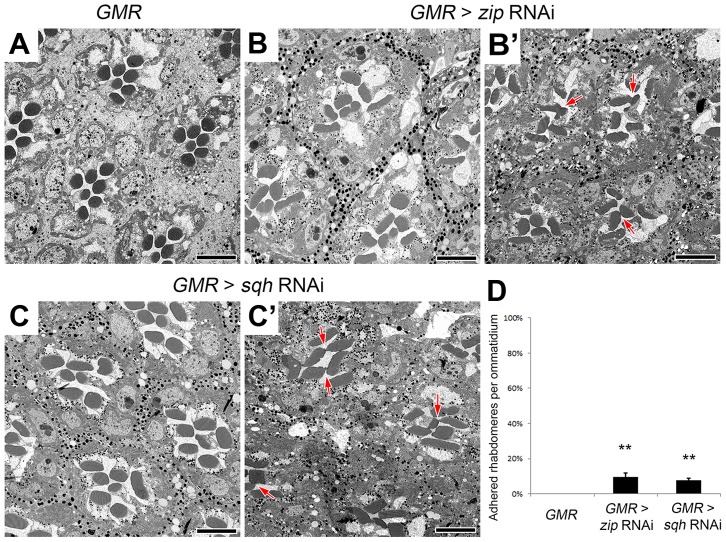
Non-muscle myosin II is required for retinal lumen formation. (A–C′) Transmission electron micrographs of adult *Drosophila* ommatidia. (A) *GMR*-Gal4/+. (B and B′) *GMR*-Gal4/+; UAS-*zip* RNAi/+. (C and C′) *GMR*-Gal4/+; UAS-*sqh* RNAi/+. (D) Quantitative analysis of rhabdomere fusion seen in (A–C′). Values represent mean ± SEM. **P<0.01 compared with *GMR*-Gal4/+. Arrows indicate adhesion between rhabdomeres. Scale bar, 5 µm.

The effect of the reduction of the actomyosin machinery components, Act5C, Zip, and Sqh, on the formation of a continuous IRS was not limited to the sensitized EP-TH background. In an *eys* or *prom* single-heterozygous mutant background rhabdomere fusion is never observed (Figure S6 A,C,F,H), but in these single-heterozygous backgrounds the reduction of *Act5C*, Zip, or Sqh resulted in the appearance of fused rhabdomeres (Figure S6). Notably, in the wild-type background in which neither EYS nor Prom level is altered, the RNAi knockdown of *zip*, *sqh* as well as mild *Act5C* knockdown is sufficient in generating juxtaposed rhabdomeres (Figure 4, Figure S7); strong knockdown of *Act5C* with the *GMR*-GAL4 leads to loss of rhabdomere structures (Figure S1 H). These results demonstrated that the actomyosin complex is not only involved in, but is also required and essential for retinal lumen formation.

### Regulation of the actomyosin machinery during retinal lumen formation

**Figure 5:**
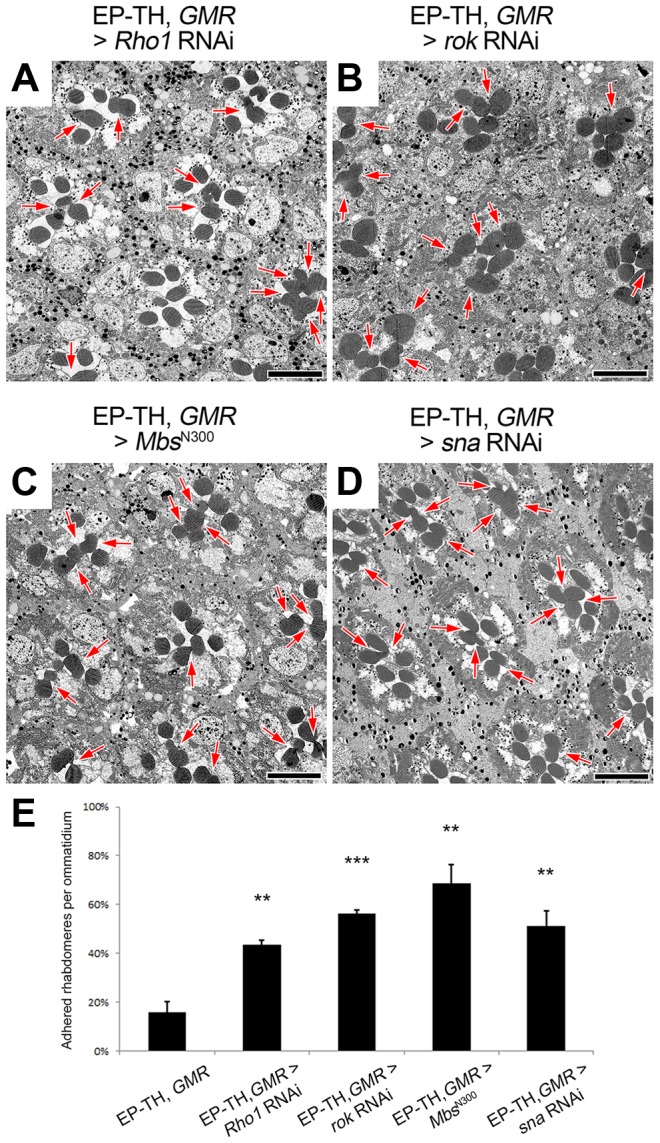
Regulators of Sqh are involved in retinal lumen formation. (A–D) Transmission electron micrographs of adult *Drosophila* ommatidia. (A) *eys*, *prom*, *GMR*-GAL4; UAS-*Rho1* RNAi/+. (B) *eys*, *prom*, *GMR*-GAL4/+; UAS-*rok* RNAi/+. (C) *eys*, *prom*, *GMR*-GAL4/+; UAS-*Mbs* ^N300^/+. (D) *eys*, *prom*, *GMR*-GAL4/+; UAS-*snail* RNAi/+. (E) Quantitative analysis of rhabdomere fusion seen in (A–D). Values represent mean ± SEM. *** *P*<0.001, **P<0.01 compared with *eys*, *prom*, *GMR*-Gal4/+. Arrows indicate the incomplete separation between rhabdomeres. Scale bar, 5 µm.

If the actomyosin machinery was a key element in the formation of the retinal lumen we should find common phenotypes among the potential upstream regulators of MyoII. To date there are more than a dozen of kinases reported to phosphorylate and activate MyoII regulatory light chain in invertebrate and vertebrate model organisms (reviewed in [40]). Fortuitously, our assay permitted a screening of potential regulators in *Drosophila* photoreceptor cells (Table S1). From our limited screen we found three candidates that when knocked-down were capable of enhancing the rhabdomere fusion in the EP-TH background: Rho-kinase (Rok) [51], and the upstream activator of Rok, the small GTPase Rho1 [52], and the upstream transcriptional regulator Snail [53] (Figure 5 A,B,D,E). However, Rok not only activates Sqh through direct phosphorylation, it also indirectly activates Sqh by inhibiting its inhibitor, the Myosin binding subunit (Mbs) of the myosin light chain phosphatase complex [54]. To test the possibility that the regulation of retinal lumen formation involves Mbs-mediated Sqh dephosphorylation, we reasoned that the overexpression of a constitutively active form of Mbs (Mbs^N300^), that lacks the Rok regulatory target site [43] would result in an increase in rhabdomere fusion in the EP-TH background. With the expression of Mbs^N300^, we observed an increase in rhabdomere fusion (Figure 5 C,E), suggesting Mbs is a negative regulator for actomyosin contraction in *Drosophila* retina. Jointly, these results indicated that in *Drosophila* photoreceptor cells, Sqh was positively regulated by Rok and Rho1, and negatively regulated by Mbs.

### The actomyosin machinery localizes apically and is required for proper rhabdomere spacing

**Figure 6:**
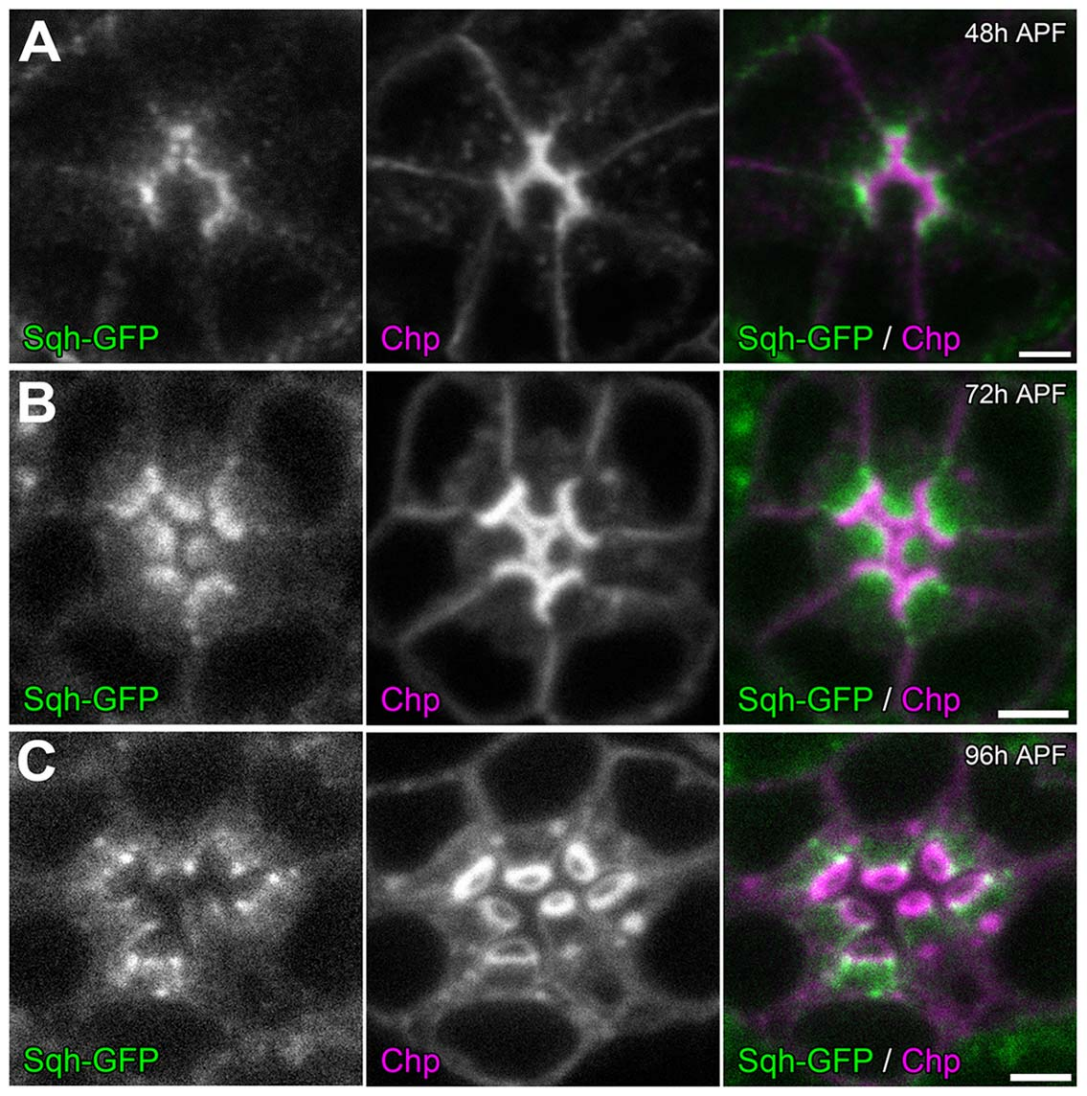
Sqh localization during retinal lumen formation. Confocal immunofluorescence micrographs of +/+; *sqh*-GFP/*sqh*-GFP (*sqh*-GFP in green) ommatidium co-stained with Chaoptin (Chp, magenta) at: (A) 48 h APF. (B) 72 h APF. (C) 96 h APF. Chaoptin labels the developing rhabdomeres.

To investigate how the actomyosin machinery might contribute to retinal lumen formation, we first determined the sub-cellular localization of MyoII. Utilizing a GFP tagged Sqh under the transcriptional control of its native promoter [55], we observed that Sqh-GFP was localized to the apical cytosol of photoreceptor cells just basal to the developing rhabdomeres (Figure 6), which is consistent with Zip antibody staining pattern in *Drosophila* photoreceptors reported previously [56]. This localization pattern of Sqh was not dependent upon an extracellular matrix, since Sqh-GFP localized normally in the *eys* null (Figure S8 A,B) and the *prom* null background (Figure S8 C,D).

**Figure 7:**
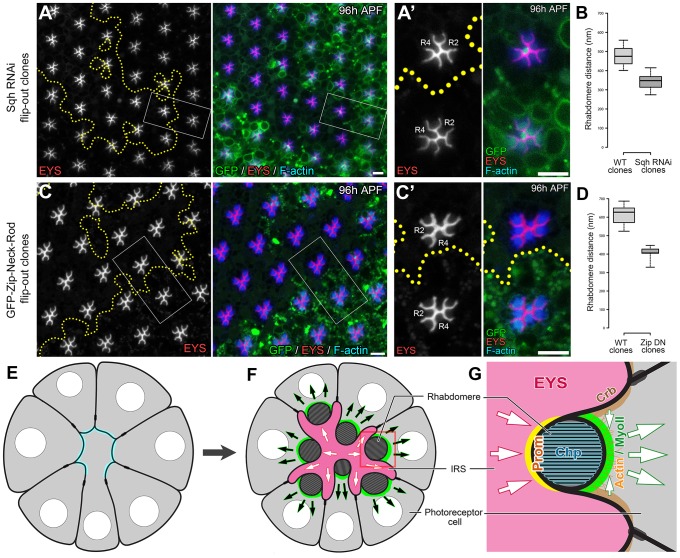
Actomyosin contraction is required for increasing the width of the retinal lumen. (A) RNAi knockdown of Sqh in the flip-out clones. *hs*-*flp*/+; *GMR*>*w* ^+^ STOP>Gal4/+; UAS-*sqh* RNAi/UAS-*mCD8*-GFP. (C) Overexpression of a dominant-negative form of Zip in flip-out clones. *hs*-*flp*/+; *GMR*>*w* ^+^ STOP>Gal4/+; UAS-GFP-*zip*-Neck-Rod/UAS-*mCD8*-GFP. GFP (green) marks the cells expressing the RNAi or dominant negative constructs, EYS (red) labels the retinal lumen, and F-actin (blue) labels the rhabdomeres. (A, C) Low magnification view. (A′, C′) A magnified view of the highlighted areas. (B, D) Box and whisker plot of the distance between the R2 and the R4 rhabdomeres of the ommatidia shown in (A) (n = 31) and (C) (n = 18), respectively. Mosaic ommatidium containing both wildtype and mutant cells were not quantified for the box plot. Boxes extend from 25^th^ to 75^th^ percentile, with a line at the median. Whiskers extend to the most extreme values (Spear style). (E–G) Model for *Drosophila* retinal lumen formation. Arrows indicate direction of forces. Scale bar, 5 µm.

The apical localization pattern of MyoII predicts that the potential contraction may act on the apical membranes. To detect potential defects in retraction of the apical membrane, we investigated whether the distance between apical rhabdomere membranes was altered upon the reduction of MyoII in a wild-type background. We generated *sqh* RNAi (Figure 7 A) and *zip* dominant-negative (GFP-Zip-Neck-Rod) (Figure 7 C) flip-out clones and measured the distance between the apical rhabdomeres. In particular we measured the width of the IRS between the apical membranes of rhabdomeres R2 and R4, two diametrically opposing rhabdomeres. The distance between apical rhabdomere membranes, which is equal to the width of the retinal lumen, was visualized by EYS staining. Our assay demonstrated that the lumen width decreased by 28% in *sqh* RNAi flip-out clones and by 33% in GFP-*zip*-Neck-Rod flip-out clones compared with their neighboring wild-type clones (Figure 7 A–D). These results are consistent with a hypothesis that the actomyosin machinery generates a contraction force at the apical photoreceptor cell membranes to pull the apical membranes inward to initiate and expand the retinal lumen (Figure 7 E–G). We also tested other aspects of cell morphology, and we found that this apically localized actomyosin machinery is not sufficient to induce a whole cell size change in photoreceptors nor does reduction of MyoII affect the overall extension of the rhabdomeres (Figure S9). However, we also noted changes in rhabdomere width, oblong rhabdomeres, with the knockdown of the actomyosin machinery but this phenotype did not correlate with the enhanced rhabdomere adhesion observed (Figure S10).

### Temporal sequence of contraction, secretion, and adhesion

**Figure 8:**
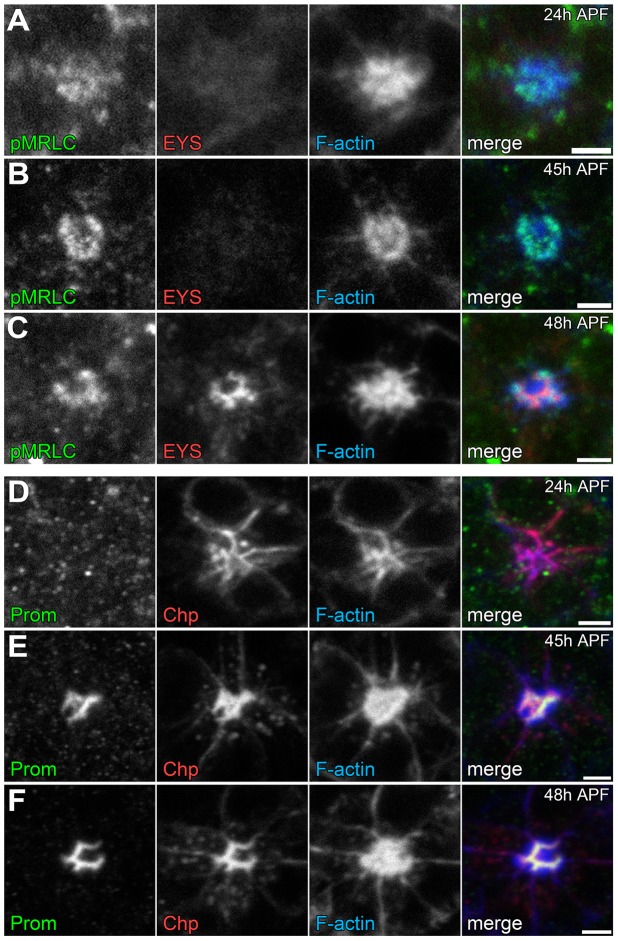
Temporal profile of the coordination of actomyosin contraction, steric hindrance of adhesion, and secretion of an extracellular matrix. (A–F) Confocal immunofluorescence micrographs of wild-type ommatidium. (A–C) Ommatidium stained with phospho-Sqh (pMRLC, green), EYS (red), and F-Actin (blue) at: (A) 24 h APF. (B) 45 h APF. (C) 48 h APF. (D–F) Ommatidium stained with Prominin (Prom, green), Chaoptin (Chp, red), and F-Actin (blue) at: (D) 24 h APF. (E) 45 h APF. (F) 48 h APF. Scale bar, 2 µm.

**Figure 9:**
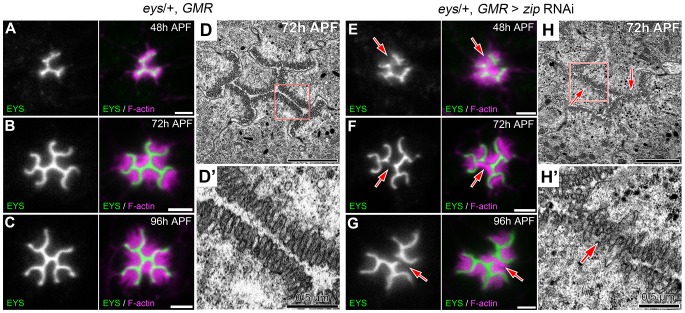
Defects in apical membrane separation are detected early and maintained throughout development. (A–C, E–G) Confocal immunofluorescence micrographs of (A–C) *eys*, *GMR*-Gal4/+ ommatidium and (E–G) *eys*, *GMR*-Gal4/+; UAS-*zip* RNAi/+ ommatidium co-stained with EYS (green) and F-actin (magenta) at (A,E) 48 h APF. (B,F) 72 h APF. (C,G) 96 h APF. (D,H) Transmission electron micrograph of (D,D′) *eys*, *GMR*-Gal4/+ ommatidium and (H,H′) *eys*, *GMR*-Gal4/+; UAS-*zip* RNAi/+ ommatidium at 72 h APF, (D′,H′) represents high magnification of boxed region in (D) or (H), respectively. Arrows denote juxtaposed apical rhabdomere membranes. Scale bar, (A–D, E–H) 2 µm, (D′,H′) 0.5 µm.

Temporally, we know that the photoreceptor cell apical membranes are juxtaposed to each other as early as 24 h APF [30] and as expected the adhesive membrane protein Chaoptin was detected on the apical surface linking the membranes together (Figure 8 D). Concurrently, we also observed not only an accumulation of F-actin at the apical surface but also the phosphorylated form of Sqh (Figure 8 A). In contrast, at 24 h APF, neither Prom nor EYS was detected on the apical surface (Figure 8 A,D). Subsequently, as the apical membranes initiate their transformation we observed the accumulation of Prom at 45 h APF on the apical surface (Figure 8 E,F) only then followed by the appearance of EYS at 48 h APF (Figure 8 B,C). Consistent with the early localization of MyoII to the apical surface, we can detect MyoII-induced rhabdomere fusion as early as 48 h APF (Figure 9); without MyoII knockdown, the *eys* heterozygous mutant alone does not lead to rhabdomere fusion (Figures S6 C). TEM analysis at 72 h clearly demonstrated the interlocking of microvilli between rhabdomeres of different photoreceptors (Figure 9 H). These data indicated that the critical developmental role of the actomyosin machinery was occurring prior to 48 h APF.

## Discussion

### *Drosophila* retinal lumen formation: a process of chord hollowing initiated by actomyosin machinery

There are several morphological strategies to generate luminal spaces (reviewed in [2], [3]). Chord hollowing is the process in which cells create a de novo lumen between their apical domains, as exemplified by Madin-Darby canine kidney (MDCK) cysts [57]. The process of chord hollowing best describes the mechanism observed in each *Drosophila* ommatidium. In *Drosophila*, the encasing of the photoreceptors by the overlying cones cells results in the apical membranes of the eight photoreceptors cells to rotate 90 degrees inward and in the end are now juxtaposed to each other [30]. Subsequently, the depositing of an extracellular matrix between the apical domains generates the retinal lumen, the IRS [16], [17]. As described lumen formation involves a few common design principles [1], [2] and with respect to chord hollowing the critical step being how space is initiated between adherent cells.

Initially, our genetic analysis of mutations for Prom, a glycosylated surface protein, and EYS, a secreted extracellular protein [17], strengthened the notion that there are molecules that provide anti-adhesive properties and the concurrent depositing of an extracellular matrix or osmotic pressure results in the permanent separation of the membranes. In *Drosophila* photoreceptors, Prom has the potential to act as an anti-adhesive molecule as described for Mucin1 and members of the CD34 family of proteins (reviewed in [2]). Prom is a five-transmembrane protein with two large extracellular loops containing a minimum of four sites that are N-glycosylated [18]. Prom is present on the apical surface before secretion of the extracellular matrix and in combination with EYS provides a barrier to prevent interactions between the adhesive molecule Chp [17], [20], [22]. Nonetheless, the exact mechanism of its anti-adhesive properties remains ambiguous. We cannot separate the role of N-glycosylation from proper trafficking of Prom to the membrane [18]. Furthermore we have not been able to confirm whether Prom's interaction with EYS is essential for membrane separation or whether the anti–adhesive properties of Prom is sufficient to prevent the interaction of Chaoptin between rhabdomeres as long as a force is supplied to keep the membranes apart. With respect to the force, the secretion of EYS fulfills this role. Not only is the separation of the membranes and formation of the retinal lumen dependent on the presence of EYS [16], [17] but alteration of the amount of EYS is sufficient to modulate the diameter of the lumen [17].

Nevertheless, even with these two well-defined parameters, anti-adhesion and secretion, numerous questions remain about membrane separation. For instance, do the photoreceptor cells themselves generate an active force for separating the rhabdomere membranes? Thus our sensitized EP-TH genetic background provided an opportunity to not only address potential questions related to the regulation of Prom and EYS but also reveal additional mechanisms for retinal lumen formation. Here our results demonstrated that the specific reduction in the genetic dosage of *Act5C* increased the likelihood that the apical membranes remaining juxtaposed. The reduction of *Act5C* genetic dosage did not affect rhabdomere morphogenesis, the trafficking of components to the apical membrane, or secretion of EYS but rather revealed the critical role of actomyosin machinery in separating the apical membranes; the reduction of the myosin heavy chain, Zip, and the myosin regulatory light chain, Sqh, phenocopied the results obtained with *Act5C*. In light of the fact that the loss of actomyosin machinery results in rhabdomere fusion in the presence of wild-type levels of Prom and EYS demonstrates that the role of the actomyosin machinery is not an accessory process but a required mechanism for retinal lumen formation.

Thus all together our data advocate a temporal framework for initiation of the retinal luminal space (Figure 7 E–G). Based on our results, the accumulation of the actomyosin complex on the apical surface occurs prior to the secretion of an extracellular matrix. The combination of MyoII and the actin meshwork generates a contractile force that pulls the apical membrane towards the center of the photoreceptor cell. Furthermore, the polarized localization of MyoII, rather than a circumferential cable pattern or a dispersed pattern, suggested that in photoreceptor cells the actomyosin contraction may not be a “purse string” or a “ratchet-like” mechanism which contracts the cell from the periphery to the center, as reported in vertebrate neurulation and in *Drosophila* mesoderm invagination (see review [38]). We propose that together with the subsequent accumulation of Prom, the actomyosin contraction provides the necessary separation and weakening of inter-apical membrane interactions. Lastly, the secretion of EYS and its interaction with Prom provides an additional separation force and permanent barrier to prevent the adhesive properties of Chp from interacting between rhabdomeric membranes, thus fully establishing and stabilizing the retinal lumen space.

### Comparison of actomyosin contraction in lumen formation and tissue morphogenesis

Whether the role of actomyosin contraction is limited to *Drosophila* retinal lumen formation or chord hollowing remains to be tested. Nonetheless, actomyosin contraction has been implicated in other luminal systems and is likely another core process required for lumen formation. For example, during murine vascular lumen formation, MyoII fails to localize to the apical cell membrane upon pharmacological inhibition of ROCK or its upstream activator vascular endothelial growth factor A, and the vascular tubes formed to a lesser extent [58]. Furthermore, the actomyosin contraction observed in *Drosophila* photoreceptors may also extend to vertebrate ciliary photoreceptors. Previous work has demonstrated the functional conservation of EYS and Prom among rhabdomeric and ciliary photoreceptors [18]. Interestingly, both Prominin1 and MyoII localize to the basal region of the outer segment where the nascent discs are formed in mammalian photoreceptors [59]–[61]. Therefore, one possibility is that actomyosin contraction is involved in the intra-cellular disk lumen formation. Specifically, actomyosin contraction might be required to pull the plasma ciliary membrane inwards to form and morphologically flatten the nascent discs [62], [63]. Overall, utilization of *Drosophila* retinal lumen as a model tissue, in particular our EP-TH sensitized genetic background, to perform unbiased screens will further define the mechanisms for activation of the actomyosin machinery and elucidate mechanisms for lumen formation and regulation.

## Materials and Methods

### *Drosophila* stocks and clonal analysis

All crosses and staging were performed at 23°C unless otherwise noted. *Drosophila* stocks used in this study include: *prom* ^1^, *eys* ^1^ [17], UAS-GFP-*zip*, UAS-GFP-*zip*-Neck-Rod (Dr. D. Kiehart), UAS-*Mbs* ^N300^ (Dr. J. Treisman), *sqh*-GFP (Dr. A. Martin), *GMR*>*w* ^+^ STOP>Gal4 (Dr. C. Desplan), and UAS-*sqh* ^E20E21^ (Dr. M. Birnbaum). The following fly stocks were obtained from the Bloomington Drosophila Stock Center: *w^1118^*, *GMR*-Gal4, UAS-*mCD8*-GFP, *Act5C* ^G0245^, *Act5C* ^G0009^, *Act5C* ^G0010^, *Act5C* ^G0025^, *Act5C* ^G0177^, *zip* ^1^, *sqh* ^AX3^, Df(1)ED6829, Df(1)ED418, Df(2R)ED1484, Df(2R)ED3791, Df(3L)BSC223, Df(3R)ED5613, Df(3R)ED5705, UAS-*zip* RNAi (TRiP #HMS01618), UAS-*sqh* RNAi (TRiP #HMS00437), UAS-*Rho1* RNAi (TRiP #JF02809), UAS-*rok* RNAi (TRiP #JF03225), UAS-*crb* RNAi (TRiP #JF02777), UAS-*Act5C* RNAi (TRiP #HMS02487), UAS-*atg1* RNAi (TRiP #JF02273, and #GL00047), UAS-*cta* RNAi (TRiP #JF01607, and #HMS02362), UAS-*fog* RNAi (TRiP #GL00529), UAS-*mist* RNAi (TRiP #HMS02327), UAS-*RhoGEF2* RNAi (TRiP #HMS01118), UAS-*sna* RNAi (TRiP #HMS01252), UAS-*SNF1A* RNAi (TRiP #JF01951, #HMS00362, and #GL00004), UAS-*sqa* RNAi (TRiP #JF02277), UAS-*Strn-Mlck* RNAi (TRiP #JF02278, #JF02170, and #HMS01665), UAS-*T48* RNAi (TRiP #HMS02248), and UAS-*twi* RNAi (TRiP #JF02003, and #HMS01317). *Pph13*-Gal4 was generated by inserting the immediate upstream 1.6 kb of genomic DNA extending from the first coding Methionine of the *Pph13* locus into pCHS-GAL4. *prom*-Gal4 was generated by inserting the immediate upstream 3.6 kb of genomic DNA extending from the first coding Methionine of the *prominin* locus into pCHS-GAL4.

To generate flip-out clones, UAS-*zip* RNAi, UAS-GFP-*zip*-Neck-Rod, or UAS-*sqh* RNAi males were crossed with *hs*-Flp; *GMR*>*w* ^+^ STOP>Gal4; UAS-*mCD8*-GFP females, and the 24 h–48 h larvae were subject to 1 h heatshock at 37°C and then returned to 23°C to generate clones. *GMR* promoter starts to drive Gal4 expression in photoreceptors of flip-out clones starting from the third instar larval stage. Clones were marked by the presence of *mCD8*-GFP. To generate MARCM clones, *sqh* ^AX3^, Frt19A; *GMR*-Gal4 females were crossed with *hs*-Flp, *tub*-Gal80, Frt19A; UAS-*mCD8*-GFP males, and the third instar larvae were subject to 1 h heatshock at 37°C and then returned to 23°C. Mutant clones were marked by the presence of *mCD8*-GFP.

### Transmission electron microscopy, immunofluorescence staining, and imaging

*Drosophila* eye samples were prepared for transmission electron microscopy (TEM) as previously described [17]. All crosses were maintained at 23°C and adult heads were fixed within 8 h after eclosion. Standard fixation and staining protocols were used for immunofluorescence staining. Briefly, pupal retinas were staged at 23°C, dissected in PBS, and fixed in PBS containing 4% formaldehyde for 10 min (24 h, 45 h, and 48 h APF pupae, as well as 72 h APF pupae for anti-Prom staining) or 40 min (72 h and 96 h APF pupae). The primary antibodies used were: mouse anti-EYS (mAb 21A6, 1∶50, Developmental Studies Hybridoma Bank) [17]; rabbit anti-Prom (1∶100) [17]; rat anti-Crb (1∶400, Dr. E. Knust) [64]; rabbit anti-Zip (#656, 1∶400, Dr. D. Kiehart) [65]; guinea pig anti-Sqh (GP#21, 1∶400, Dr. D. Kiehart) [66]; mouse anti–phospho–Sqh (Ser19, analogous to *D. melanogaster Sqh* Ser21) (pMRLC, 1∶100, Cell Signaling Technology); mouse anti-Chp (mAb 24B10, 1∶100, Developmental Studies Hybridoma Bank) [19]; mouse anti-Na^+^ K^+^ ATPase alpha subunit (a5, 1∶100, Developmental Studies Hybridoma Bank) [29]; rabbit anti-phospho-Moesin (pMoe) (mAb 41A3, 1∶100, Cell Signaling). Rhodamine (1∶200) or Alexa Fluor 647 (1∶50) conjugated phalloidin (Life Technologies) was used for the detection of F-actin. The FITC or RX conjugated secondary antibodies (1∶200) were obtained from Jackson ImmunoResearch Laboratories. Confocal images were taken on a Leica TCS SP5 and TEM was performed with a JOEL 1010, and all pictures were processed in Adobe Photoshop.

## Supporting Information

Figure S1 Reduction of *Act5C* dosage enhances rhabdomere adhesion. (A–G) Transmission electron micrographs of adult *Drosophila* ommatidia. (A) *eys*, *prom*/+. (B) Df(1)ED418/+; *eys*, *prom*/+. The deficiency removes genomic region 5C7-5E4. (C) *Act5C* ^G0009^/+; *eys*, *prom*/+. (D) *Act5C* ^G0010^/+; *eys*, *prom*/+. (E) *Act5C* ^G0025^/+; *eys*, *prom*/+. (F) *Act5C* ^G0177^/+; *eys*, *prom*/+. (G) *eys*, *prom*/+; *prom*-Gal4/UAS-*Act5C* RNAi. (H) *eys*, *prom*, *GMR*-GAL4/+; UAS-*Act5C* RNAi/+. Arrows indicate the incomplete separation between rhabdomeres. Scale bar, 5 µm.
(TIF)

Figure S2 Rhabdomere fusion is specific to the reduction in *Act5C*. (A–F) Transmission electron micrographs of adult *Drosophila* ommatidia of (A) *eys*, *prom/+* with deficiencies that remove one of the two copies of: (B) *Act42A*, (C) *Act57B*, (D) *Act79B*, (E) *Act87E*, (F) *Act88F*. Arrows indicate the incomplete separation between rhabdomeres. Scale bar, 5 µm.
(TIF)

Figure S3 Reduction of *Act5C* dosage does not affect the vertical extension of the rhabdomeres. (A–D) Confocal immunofluorescence micrographs showing the vertical view of adult *Drosophila* ommatidia. (A) *w^1118^*, wild type. (B) *Act5C* ^G0245^/+. (C) *eys*, *prom*/+. (D) *Act5C* ^G0245^/+; *eys*, *prom*/+. Na^+^ K^+^ ATPase (NaK, green) labels the basolateral membranes of photoreceptor cells, and F-actin (magenta) labels the rhabdomeres. Scale bar, 10 µm.
(TIF)

Figure S4 The localization of phospho-Moesin is not affected by the reduction of *Act5C* genetic dosage. (A,B) Confocal immunofluorescence micrographs of 48 h APF *Drosophila* ommatidium. Phospho-Moesin (pMoe, green) labels the activated form of Moesin, and F-actin (magenta) labels the rhabdomeres. (A) *w^1118^*, wild type. (B) *Act5C* ^G0245^/+. Scale bar, 2 µm.
(TIF)

Figure S5 *zip* RNAi and *sqh* RNAi specifically target zipper and spaghetti squash. (A,B) Confocal immunofluorescence micrographs of *zip* RNAi flip-out clones (*hs*-*flp*/+; *GMR*>*w* ^+^ STOP>Gal4/+; UAS-*zip* RNAi/UAS-*mCD8*-GFP) of pupal retinas at 48 h APF visualizing mCD8-GFP (green), Zip (red), and F-Actin (blue). The RNAi expressing cells are marked with GFP and resulted in a reduction of Zip immunoreactivity. (C,D) Confocal immunofluorescence micrographs of *sqh* RNAi flip-out clones (*hs*-*flp*/+; *GMR*>*w* ^+^ STOP>Gal4/+; UAS-*sqh* RNAi/UAS-*mCD8*-GFP) of pupal retinas at 48 h visualizing mCD8-GFP (green), Sqh (red), and F-Actin (blue). The RNAi expressing cells are marked with GFP and resulted in a reduction of Sqh immunoreactivity. Dotted lines delineate wild-type and knockdown cells. (E–H) Transmission electron micrographs of adult *Drosophila* ommatidia of (E) *eys*, *prom*, *GMR*-GAL4/+; UAS-*zip* RNAi/UAS-*mCD8-*GFP. (F) *eys*, *prom*, *GMR*-GAL4/+; UAS-*zip* RNAi/UAS-GFP-zip. (G) *eys*, *prom*, *GMR*-GAL4/+; UAS-*sqh* RNAi/UAS-*mCD8-*GFP. (H) *eys*, *prom*, *GMR*-GAL4/+; UAS-*sqh* RNAi/UAS-*sqh* ^E20E21^. Arrows indicate the incomplete separation between rhabdomeres. Scale bar, (A,C) 30 µm, (B,D, E–H) 5 µm.
(TIF)

Figure S6 Reduction of actomyosin components results in rhabdomere fusion in *eys* or *prom* single-heterozygote background. (A–J) Transmission electron micrographs of adult *Drosophila* ommatidia of (A) *eys*/+. (B) *Act5C* ^G0245^/+; *eys*/+. (C) *eys*, *GMR*-Gal4/+. (D) *eys*, *GMR*-Gal4/+; UAS-*zip* RNAi/+, (E) *eys*, *GMR*-Gal4/+; UAS-*sqh* RNAi/+. (F) *prom*/+ (G) *Act5C* ^G0245^/+; *prom*/+. (H) *prom*, *GMR*-Gal4/+. (I) *prom*, *GMR*-Gal4/+; UAS-*zip* RNAi/+, (J) *prom*, *GMR*-Gal4/+; UAS-*sqh* RNAi/+. Arrows indicate the incomplete separation between rhabdomeres. Scale bar, 5 µm.
(TIF)

Figure S7 RNAi knockdown of *Act5C* results in rhabdomere fusion in the otherwise wild-type background. (A–D′) Transmission electron micrographs of adult *Drosophila* ommatidia of (A) *w* ^1118^. (B) *Act5C* ^G0245^/+. (C,C′) +/+; *prom*-Gal4/UAS-*Act5C* RNAi. (D,D′) *+/+; Pph13*-Gal4/UAS-*Act5C* RNAi. (C′,D′) A magnified view of the highlighted areas in (C) and (D), respectively. Arrows indicate the incomplete separation between rhabdomeres. Scale bar, (A,B,C,D) 5 µm, (C′,D′) 2 µm.
(TIF)

Figure S8 Sqh localization is not dependent on Prominin or EYS. Confocal immunofluorescence micrographs of *eys* (A,B) and *prom* (C,D) null mutant ommatidium with *sqh*-GFP (green) expression, and stained with Chaoptin (Chp, red), and F-Actin (blue) at 48 h APF (A,C) and 72 h APF (B,D). Chaoptin marks the rhabdomeres, and F-actin mainly marks the rhabdomeres but also weakly labels the rhabdomere terminal web. Scale bar, 2 µm.
(TIF)

Figure S9 Reduction of Sqh does not alter the whole cell size. (A–E) Confocal immunofluorescence micrographs of clones of cells lacking Sqh protein in (A–D) horizontal and (E) vertical optical sections. Effects of loss of Sqh by RNAi knockdown (A,B,E) or MARCM (C,D) are imaged at (A,C) 48 h APF, (B,D) 72 h APF, or (E) in adult eyes. (A,B,E) *hs*-*flp*/+; *GMR*>*w* ^+^ STOP>Gal4/+; UAS-*sqh* RNAi/UAS-*mCD8*-GFP. (C,D) *sqh* ^AX3^, Frt19A/*hs*-*flp*, *tub*-Gal80, Frt19A; *GMR*-Gal4, UAS-*mCD8*-GFP/+. In all panels, GFP marks the mutant cells. Na^+^ K^+^ ATPase (NaK, red in A–D and blue in E) labels the lateral and basal plasma membrane and F-actin (blue in A–D and red in E) labels the developing rhabdomeres. Scale bars, (A–D) 5 µm, (E) 20 µm.
(TIF)

Figure S10 Changes in rhabdomere width do not contribute to the adhesion of rhabdomeres. (A) Transmission electron micrographs of adult *eys*, *prom*, *GMR*-GAL4/+; UAS-*sqh* RNAi ommatidia. Oblong rhabdomeres are defined as rhabdomeres with their width equal to or greater than twice of their length. Arrows indicate fusion between rhabdomeres, and asterisks label oblong rhabdomeres. (B) Quantitative analysis of rhabdomere fusion in normal ommatidia and in ommatidia with oblong rhabdomere(s). Not statistically significant (NS), *P*>0.05. Scale bars, 2 µm.
(TIF)

Table S1 Tested potential actomyosin regulators via RNAi in the EP-TH genetic background.
(DOCX)
