# Supplementary figures and images for: The Actomyosin Machinery Is Required for Drosophila Retinal Lumen Formation
Source: PLoS Genet. 2014 Sep 18;10(9):e1004608. doi: 10.1371/journal.pgen.1004608 (PMC4168998; doi:10.1371/journal.pgen.1004608)

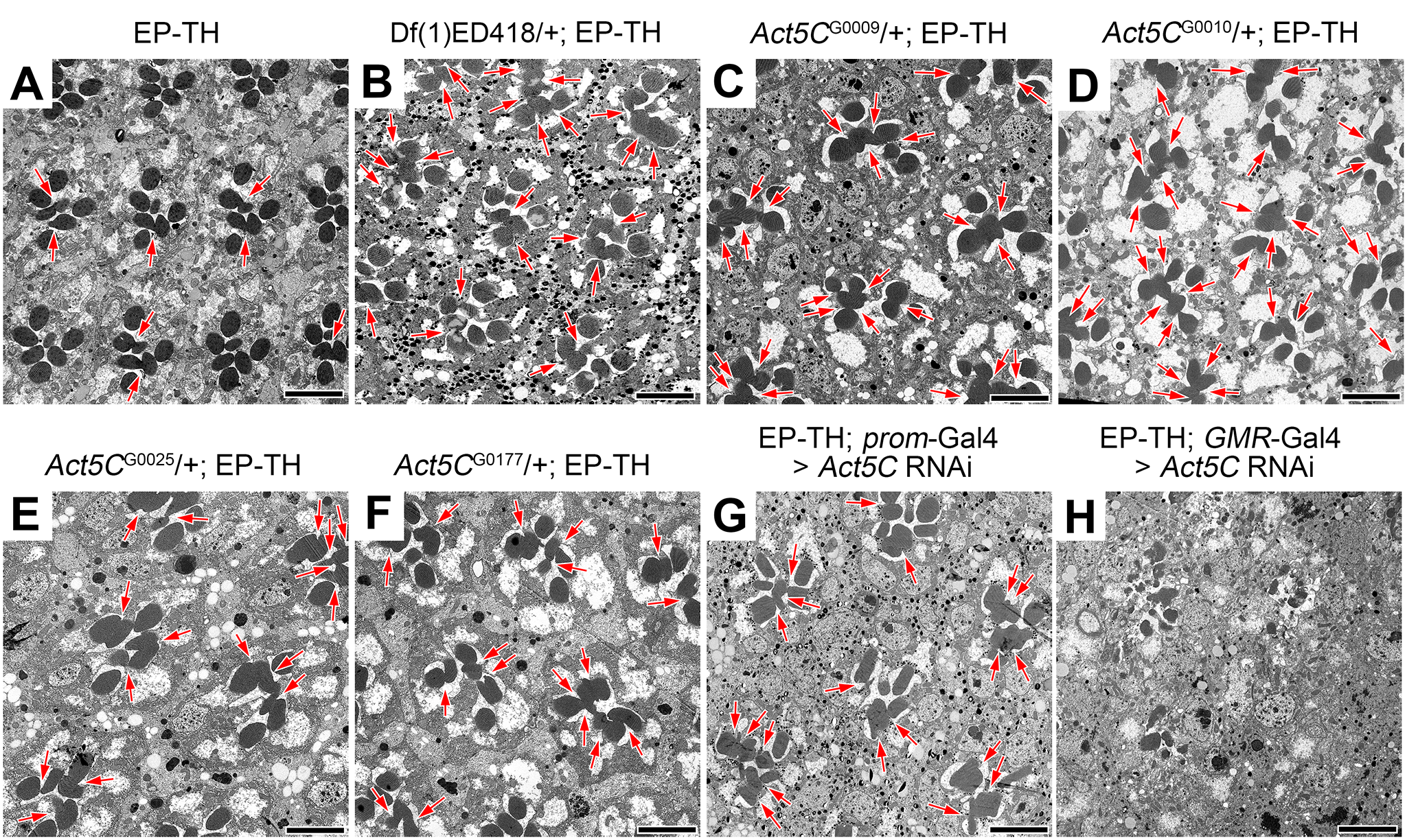

Supplement: Figure S1 — Reduction of Act5C dosage enhances rhabdomere adhesion. (A–G) Transmission electron micrographs of adult Drosophila ommatidia. (A) eys, prom/+. (B) Df(1)ED418/+; eys, prom/+. The deficiency removes genomic region 5C7-5E4. (C) Act5C G0009/+; eys, prom/+. (D) Act5C G0010/+; eys, prom/+. (E) Act5C G0025/+; eys, prom/+. (F) Act5C G0177/+; eys, prom/+. (G) eys, prom/+; prom-Gal4/UAS-Act5C RNAi. (H) eys, prom, GMR-GAL4/+; UAS-Act5C RNAi/+. Arrows indicate the incomplete separation between rhabdomeres. Scale bar, 5 µm. (TIF) [file pgen.1004608.s001.tif]

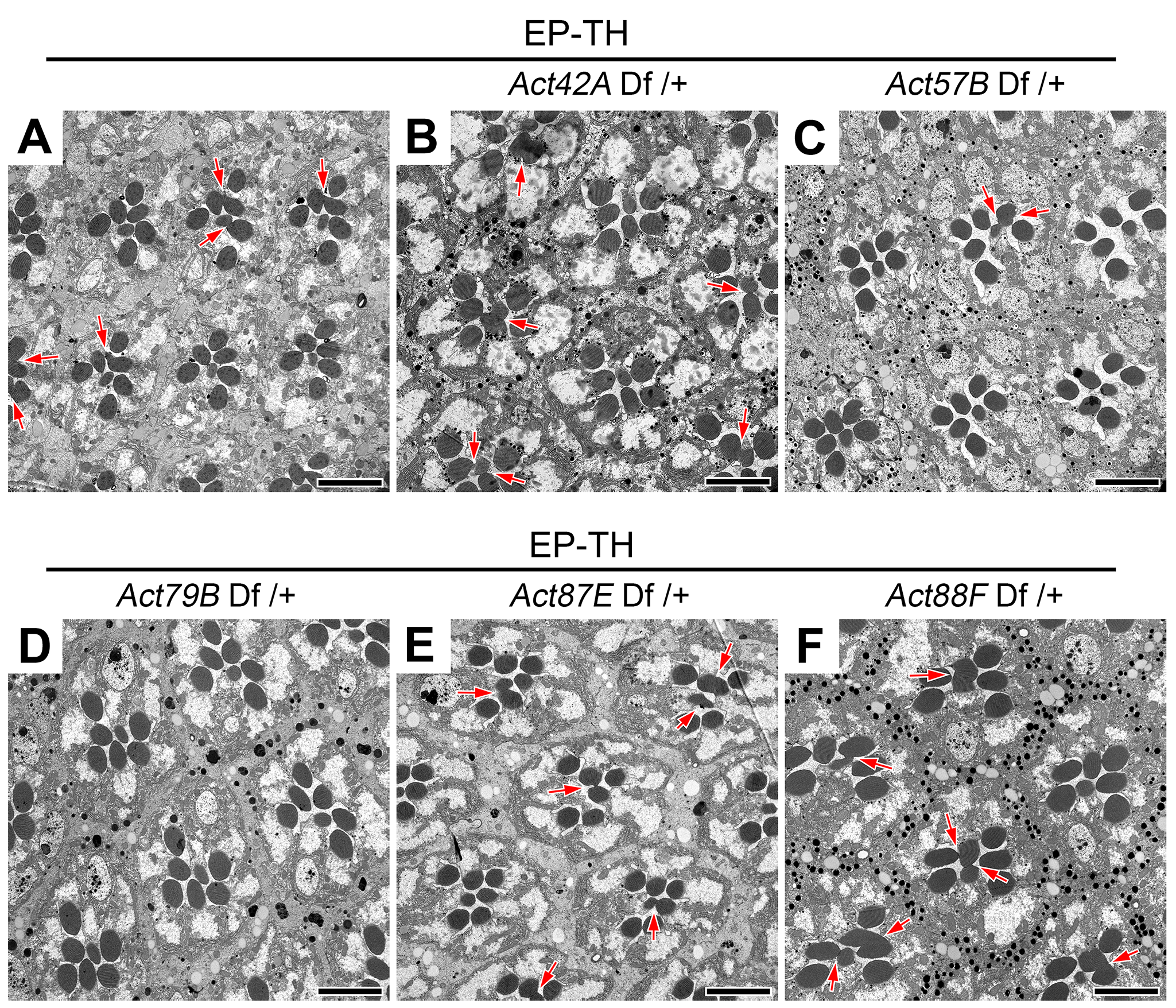

Supplement: Figure S2 — Rhabdomere fusion is specific to the reduction in Act5C. (A–F) Transmission electron micrographs of adult Drosophila ommatidia of (A) eys, prom/+ with deficiencies that remove one of the two copies of: (B) Act42A, (C) Act57B, (D) Act79B, (E) Act87E, (F) Act88F. Arrows indicate the incomplete separation between rhabdomeres. Scale bar, 5 µm. (TIF) [file pgen.1004608.s002.tif]

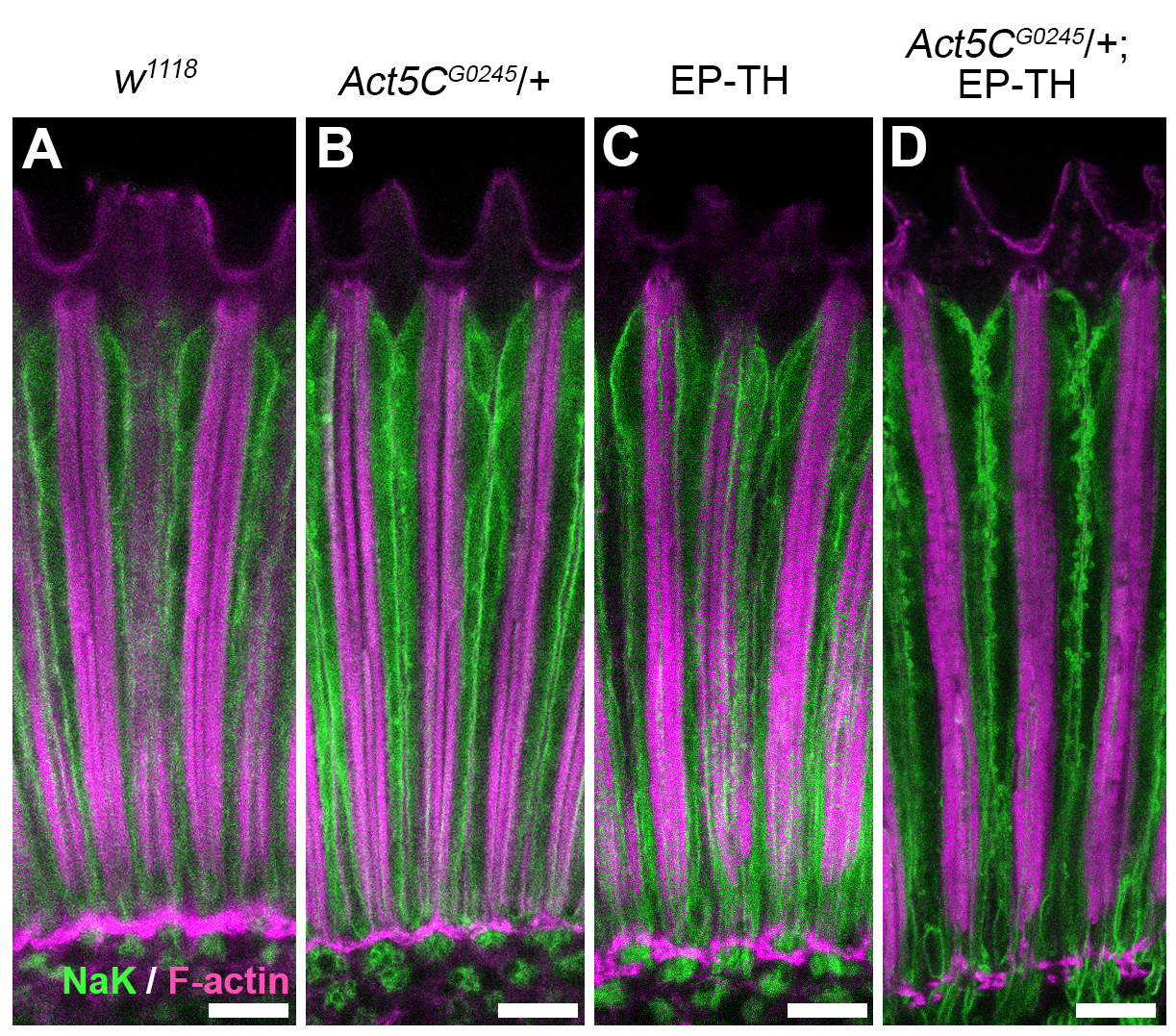

Supplement: Figure S3 — Reduction of Act5C dosage does not affect the vertical extension of the rhabdomeres. (A–D) Confocal immunofluorescence micrographs showing the vertical view of adult Drosophila ommatidia. (A) w1118, wild type. (B) Act5C G0245/+. (C) eys, prom/+. (D) Act5C G0245/+; eys, prom/+. Na+ K+ ATPase (NaK, green) labels the basolateral membranes of photoreceptor cells, and F-actin (magenta) labels the rhabdomeres. Scale bar, 10 µm. (TIF) [file pgen.1004608.s003.tif]

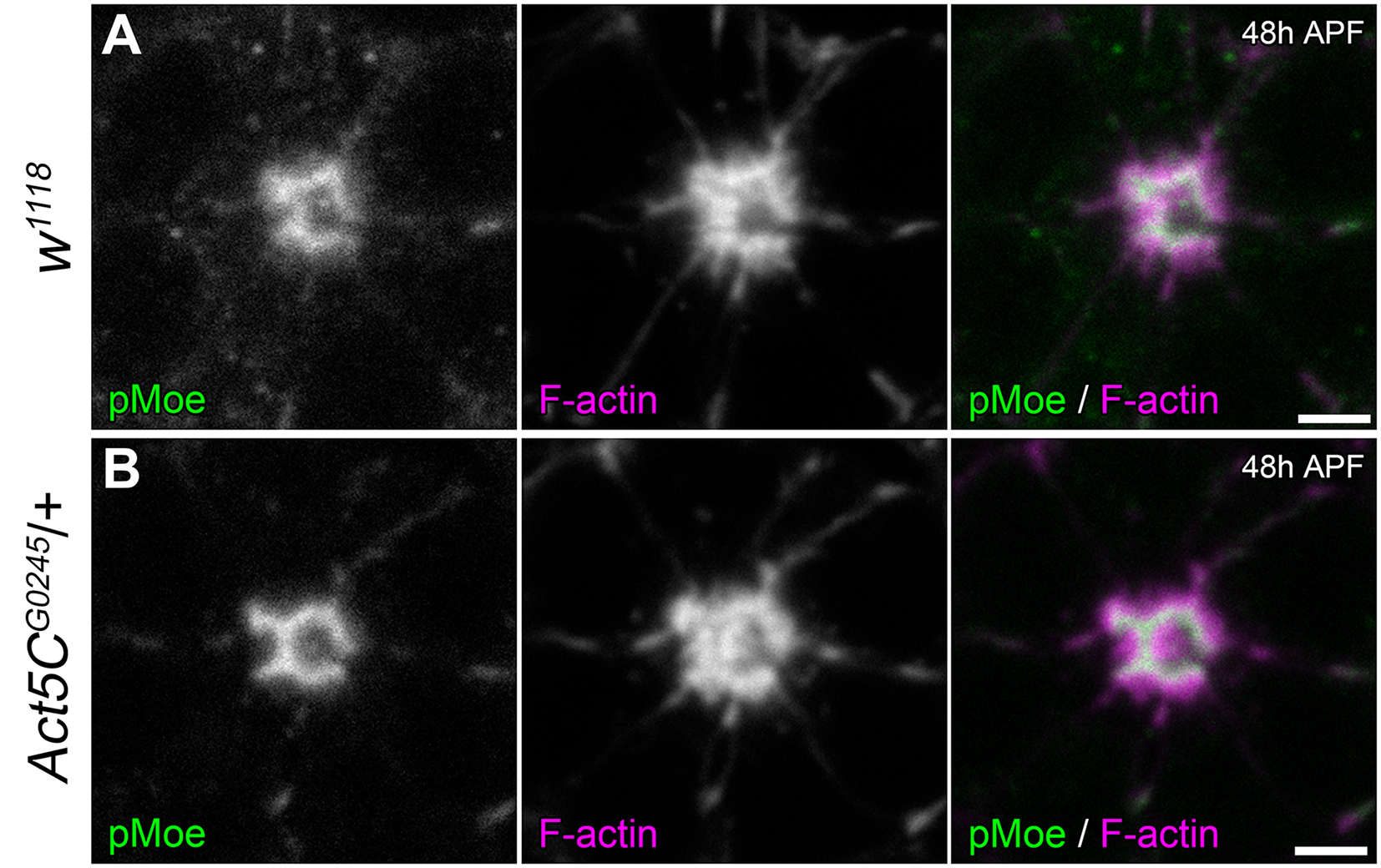

Supplement: Figure S4 — The localization of phospho-Moesin is not affected by the reduction of Act5C genetic dosage. (A,B) Confocal immunofluorescence micrographs of 48 h APF Drosophila ommatidium. Phospho-Moesin (pMoe, green) labels the activated form of Moesin, and F-actin (magenta) labels the rhabdomeres. (A) w1118, wild type. (B) Act5C G0245/+. Scale bar, 2 µm. (TIF) [file pgen.1004608.s004.tif]

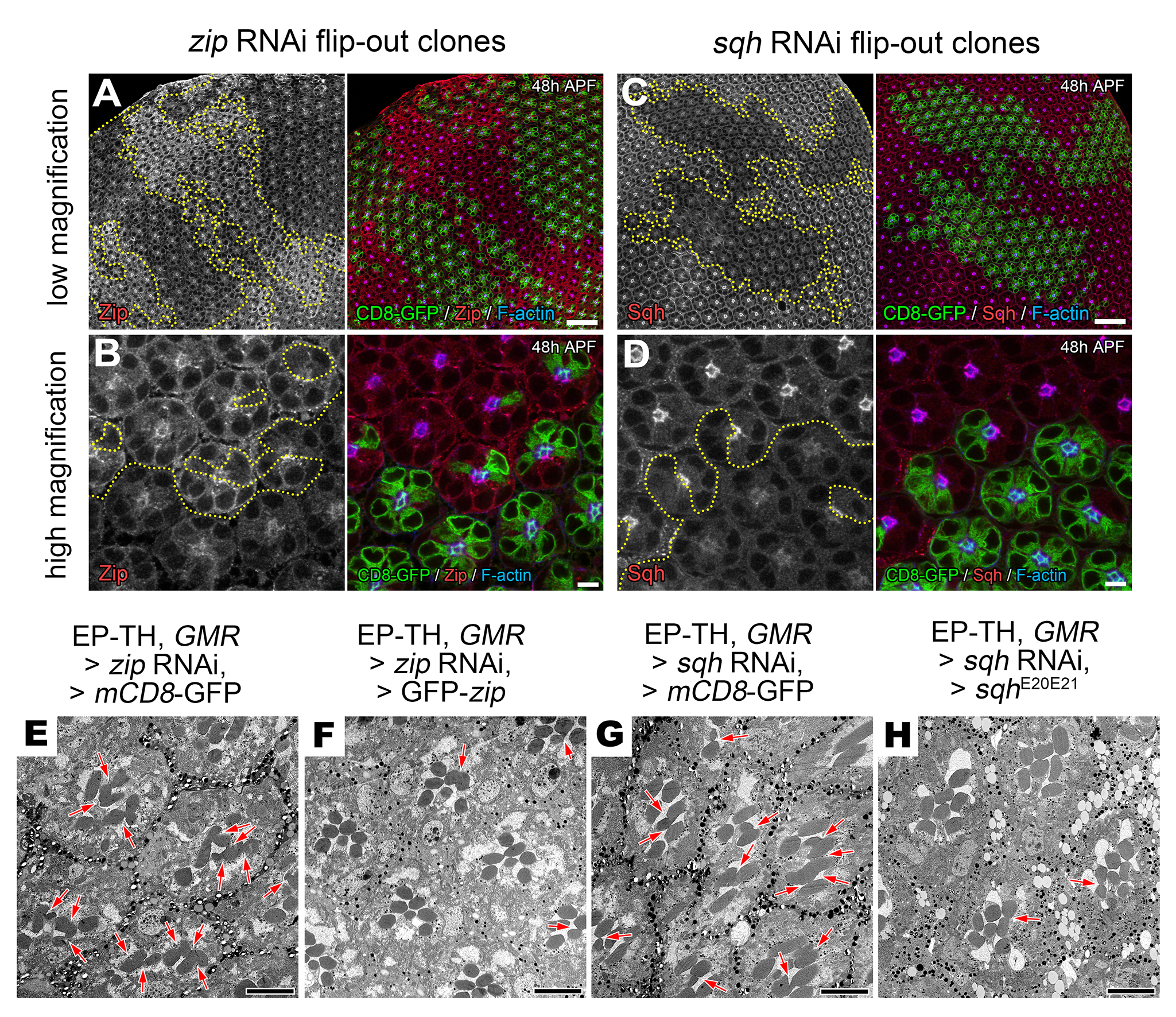

Supplement: Figure S5 — zip RNAi and sqh RNAi specifically target zipper and spaghetti squash. (A,B) Confocal immunofluorescence micrographs of zip RNAi flip-out clones (hs-flp/+; GMR>w + STOP>Gal4/+; UAS-zip RNAi/UAS-mCD8-GFP) of pupal retinas at 48 h APF visualizing mCD8-GFP (green), Zip (red), and F-Actin (blue). The RNAi expressing cells are marked with GFP and resulted in a reduction of Zip immunoreactivity. (C,D) Confocal immunofluorescence micrographs of sqh RNAi flip-out clones (hs-flp/+; GMR>w + STOP>Gal4/+; UAS-sqh RNAi/UAS-mCD8-GFP) of pupal retinas at 48 h visualizing mCD8-GFP (green), Sqh (red), and F-Actin (blue). The RNAi expressing cells are marked with GFP and resulted in a reduction of Sqh immunoreactivity. Dotted lines delineate wild-type and knockdown cells. (E–H) Transmission electron micrographs of adult Drosophila ommatidia of (E) eys, prom, GMR-GAL4/+; UAS-zip RNAi/UAS-mCD8-GFP. (F) eys, prom, GMR-GAL4/+; UAS-zip RNAi/UAS-GFP-zip. (G) eys, prom, GMR-GAL4/+; UAS-sqh RNAi/UAS-mCD8-GFP. (H) eys, prom, GMR-GAL4/+; UAS-sqh RNAi/UAS-sqh E20E21. Arrows indicate the incomplete separation between rhabdomeres. Scale bar, (A,C) 30 µm, (B,D, E–H) 5 µm. (TIF) [file pgen.1004608.s005.tif]

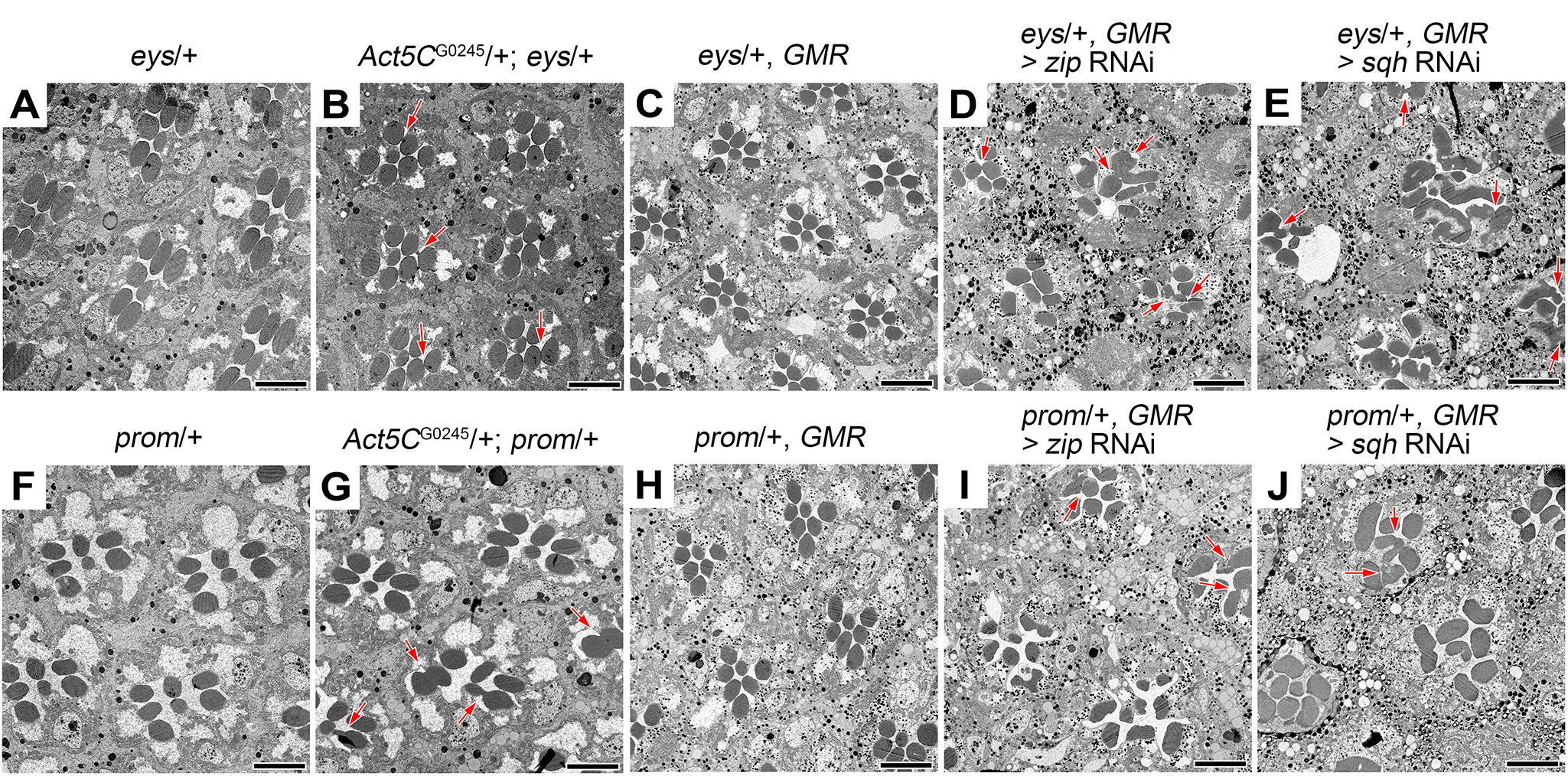

Supplement: Figure S6 — Reduction of actomyosin components results in rhabdomere fusion in eys or prom single-heterozygote background. (A–J) Transmission electron micrographs of adult Drosophila ommatidia of (A) eys/+. (B) Act5C G0245/+; eys/+. (C) eys, GMR-Gal4/+. (D) eys, GMR-Gal4/+; UAS-zip RNAi/+, (E) eys, GMR-Gal4/+; UAS-sqh RNAi/+. (F) prom/+ (G) Act5C G0245/+; prom/+. (H) prom, GMR-Gal4/+. (I) prom, GMR-Gal4/+; UAS-zip RNAi/+, (J) prom, GMR-Gal4/+; UAS-sqh RNAi/+. Arrows indicate the incomplete separation between rhabdomeres. Scale bar, 5 µm. (TIF) [file pgen.1004608.s006.tif]

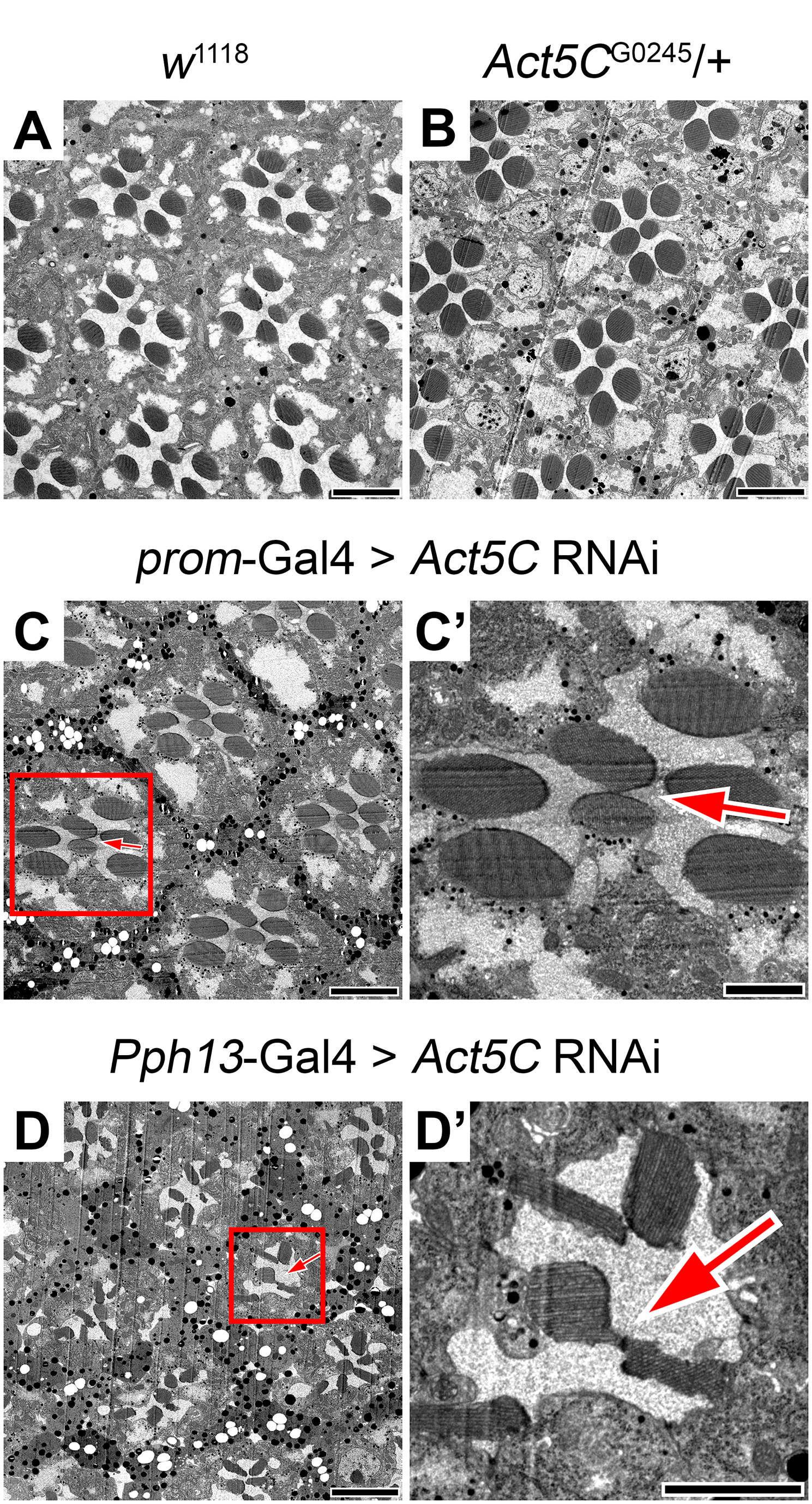

Supplement: Figure S7 — RNAi knockdown of Act5C results in rhabdomere fusion in the otherwise wild-type background. (A–D′) Transmission electron micrographs of adult Drosophila ommatidia of (A) w 1118. (B) Act5C G0245/+. (C,C′) +/+; prom-Gal4/UAS-Act5C RNAi. (D,D′) +/+; Pph13-Gal4/UAS-Act5C RNAi. (C′,D′) A magnified view of the highlighted areas in (C) and (D), respectively. Arrows indicate the incomplete separation between rhabdomeres. Scale bar, (A,B,C,D) 5 µm, (C′,D′) 2 µm. (TIF) [file pgen.1004608.s007.tif]

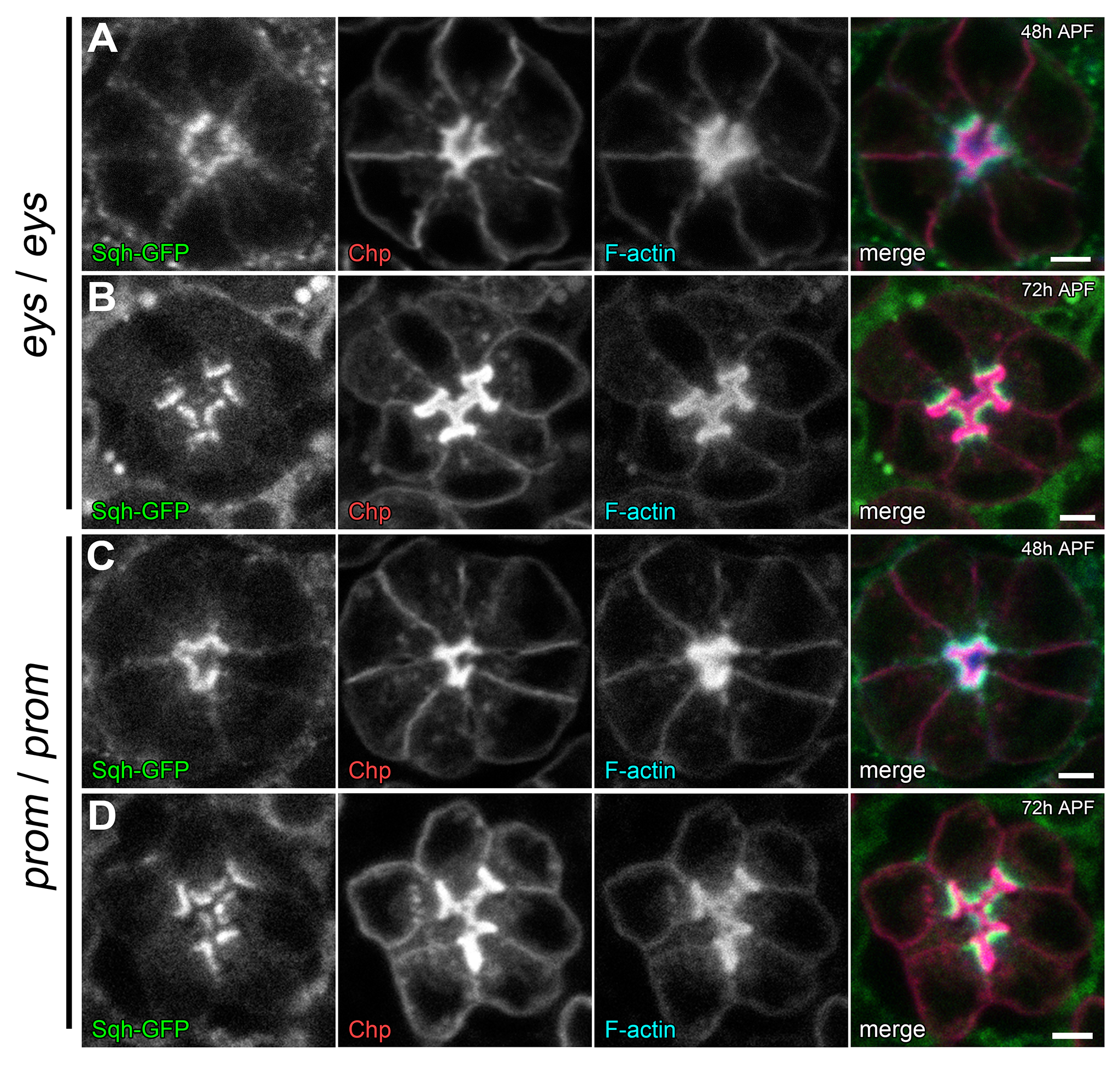

Supplement: Figure S8 — Sqh localization is not dependent on Prominin or EYS. Confocal immunofluorescence micrographs of eys (A,B) and prom (C,D) null mutant ommatidium with sqh-GFP (green) expression, and stained with Chaoptin (Chp, red), and F-Actin (blue) at 48 h APF (A,C) and 72 h APF (B,D). Chaoptin marks the rhabdomeres, and F-actin mainly marks the rhabdomeres but also weakly labels the rhabdomere terminal web. Scale bar, 2 µm. (TIF) [file pgen.1004608.s008.tif]

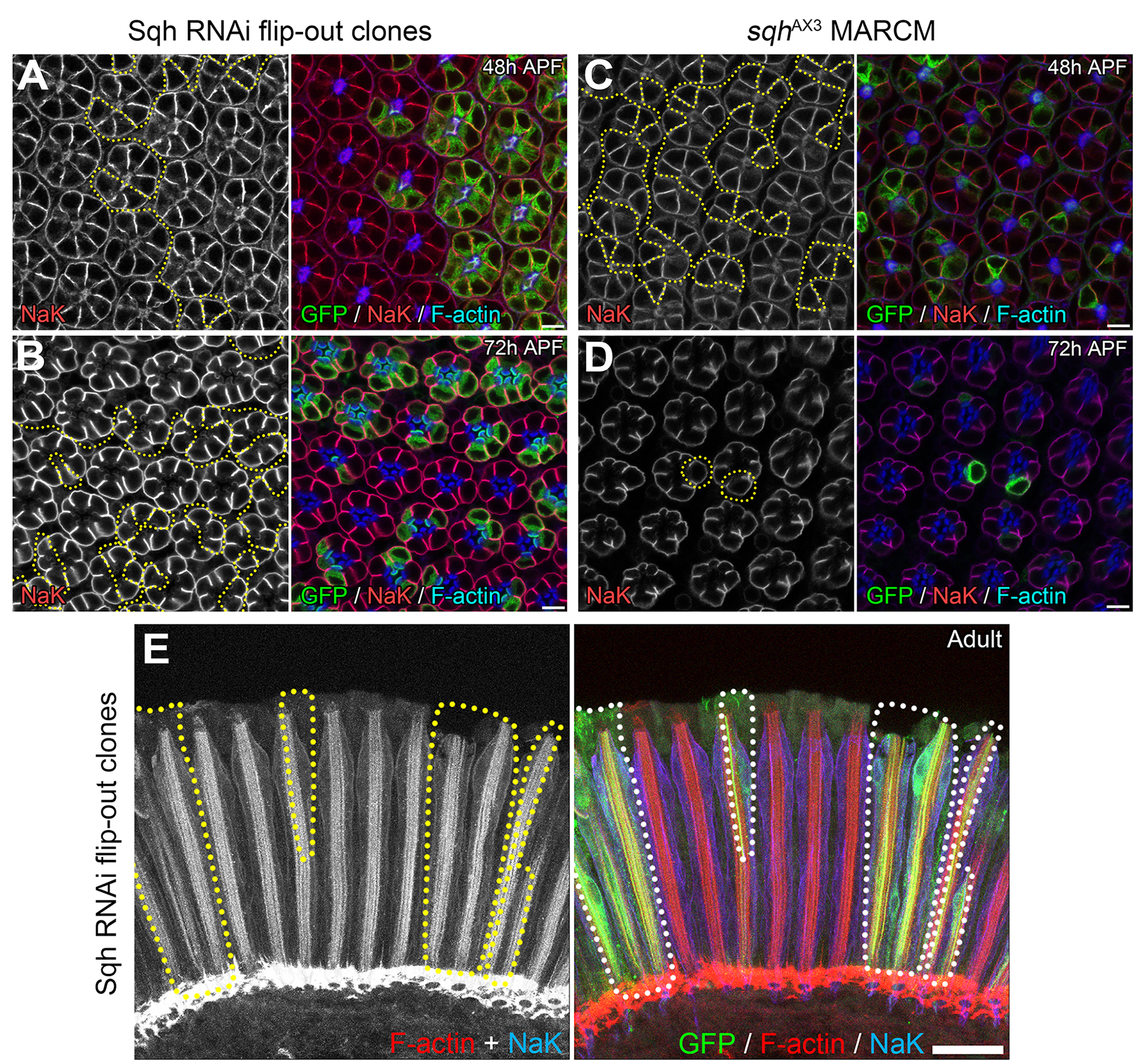

Supplement: Figure S9 — Reduction of Sqh does not alter the whole cell size. (A–E) Confocal immunofluorescence micrographs of clones of cells lacking Sqh protein in (A–D) horizontal and (E) vertical optical sections. Effects of loss of Sqh by RNAi knockdown (A,B,E) or MARCM (C,D) are imaged at (A,C) 48 h APF, (B,D) 72 h APF, or (E) in adult eyes. (A,B,E) hs-flp/+; GMR>w + STOP>Gal4/+; UAS-sqh RNAi/UAS-mCD8-GFP. (C,D) sqh AX3, Frt19A/hs-flp, tub-Gal80, Frt19A; GMR-Gal4, UAS-mCD8-GFP/+. In all panels, GFP marks the mutant cells. Na+ K+ ATPase (NaK, red in A–D and blue in E) labels the lateral and basal plasma membrane and F-actin (blue in A–D and red in E) labels the developing rhabdomeres. Scale bars, (A–D) 5 µm, (E) 20 µm. (TIF) [file pgen.1004608.s009.tif]

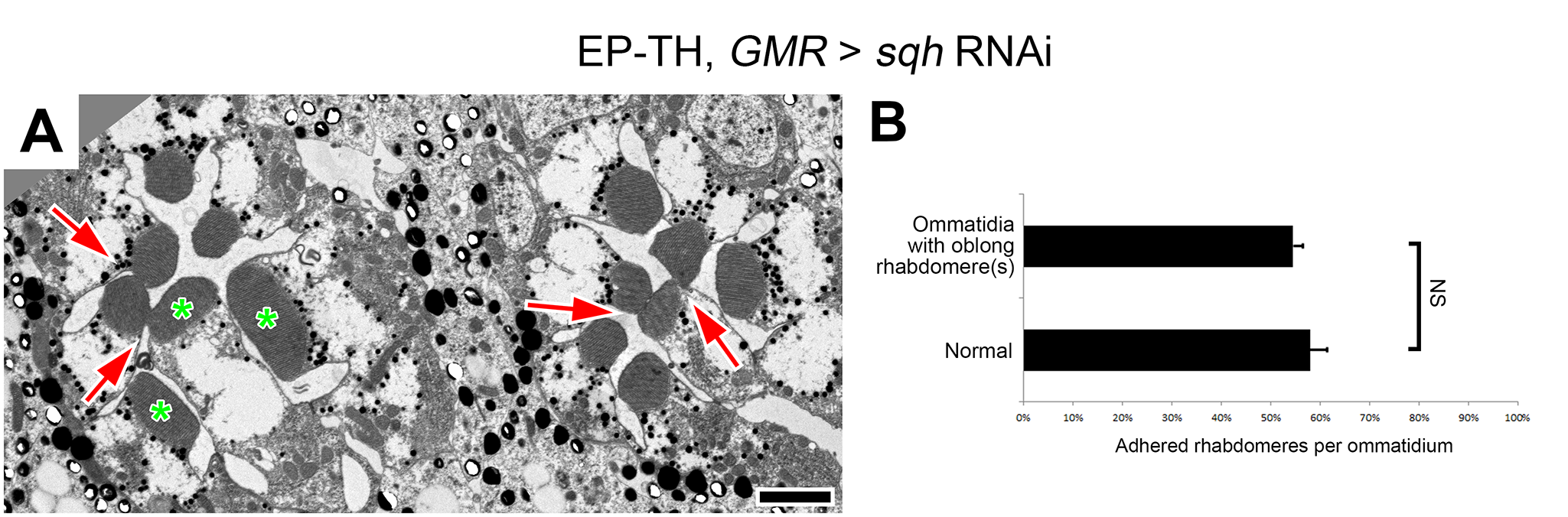

Supplement: Figure S10 — Changes in rhabdomere width do not contribute to the adhesion of rhabdomeres. (A) Transmission electron micrographs of adult eys, prom, GMR-GAL4/+; UAS-sqh RNAi ommatidia. Oblong rhabdomeres are defined as rhabdomeres with their width equal to or greater than twice of their length. Arrows indicate fusion between rhabdomeres, and asterisks label oblong rhabdomeres. (B) Quantitative analysis of rhabdomere fusion in normal ommatidia and in ommatidia with oblong rhabdomere(s). Not statistically significant (NS), P>0.05. Scale bars, 2 µm. (TIF) [file pgen.1004608.s010.tif]
