# Supplementary material for: The Actomyosin Machinery Is Required for Drosophila Retinal Lumen Formation
Source: PLoS Genet. 2014 Sep 18;10(9):e1004608. doi: 10.1371/journal.pgen.1004608 (PMC4168998; doi:10.1371/journal.pgen.1004608)
Supplement: Table S1 — Tested potential actomyosin regulators via RNAi in the EP-TH genetic background. (DOCX) [file pgen.1004608.s011.docx]

**Table S1**

| **Gene Symbol** | **Enhancement of rhabdomere adhesion by RNAi?** | **Mammalian homolog** | **Function** |
| --- | --- | --- | --- |
| *Act5C* | N/A (rhabdomere degeneration) | *ACTB / ACTG1* | NM II interacts with actin filaments and induce contraction |
| *sqh* | Yes | *MRLC* | NM II regulatory light chain |
| *zip* | Yes | *MHC* | NM II heavy chain |
| *rok* | Yes | *ROCK* | Sqh (MRLC) activator and Mbs suppressor (Amano et al., 1996; Kimura et al., 1996) |
| *SNF1A* | No | *AMPKa1* | Sqh (MRLC) activator |
| *sqa* | No | *ZIPK* | Sqh (MRLC) activator |
| *Strn-Mlck* | No | *MLCK* | Sqh (MRLC) activator |
| *atg1* | No | *Ulk1* | Sqa activator |
| *Rho1* | Yes | *RhoA* | Rok activator |
| *RhoGEF2* | No | *ARHGEF11* | Rho1 activator |
| *cta* | No | *GNA12 / GNA13* | RhoGEF2 activator |
| *T48* | No | ? | Recruiting RhoGEF2 to the apical membrane |
| *mist* | No | ? | Receptor for Fog and activator of Cta |
| *fog* | No | ? | Upstream activator of Cta through Mist |
| *sna* | Yes | (Snail family genes) | Positive regulation of Mist |
| *twi* | Mild enhancement | *TWIST1* | Positive regulator of Snail, Fog, and T48 |
| *crb* | Yes | *CRB1* | Modulating the length of stalk membrane |
